# *Arboricolonus simplex* gen. et sp. nov. and novelties in *Cadophora*, *Minutiella* and *Proliferodiscus* from *Prunus* wood in Germany

**DOI:** 10.3897/mycokeys.63.46836

**Published:** 2020-03-02

**Authors:** Steffen Bien, Ulrike Damm

**Affiliations:** 1 Senckenberg Museum of Natural History Görlitz, PF 300 154, 02806 Görlitz, Germany Senckenberg Museum of Natural History Görlitz Görlitz Germany; 2 International Institute Zittau, Technische Universität Dresden, Markt 23, 02763 Zittau, Germany Technische Universität Dresden Zittau Germany

**Keywords:** Ascomycota, Eurotiomycetes, Leotiomycetes, new taxa, phylogeny, systematics

## Abstract

During a survey on fungi associated with wood necroses of *Prunus* trees in Germany, strains belonging to the Leotiomycetes and Eurotiomycetes were detected by preliminary analyses of ITS sequences. Multi-locus phylogenetic analyses (LSU, ITS, *TUB*, *EF-1α*, depending on genus) of 31 of the 45 strains from *Prunus* and reference strains revealed several new taxa, including *Arboricolonus***gen. nov.**, a new genus in the Helotiales (Leotiomycetes) with a collophorina-like asexual morph. Seven *Cadophora* species (Helotiales, Leotiomycetes) were treated. The 29 strains from *Prunus* belonged to five species, of which *C. luteo-olivacea* and *C. novi-eboraci* were dominating; *C. africana***sp. nov.**, *C. prunicola***sp. nov.** and *C. ramosa***sp. nov.** were revealed as new species. The genus *Cadophora* was reported from *Prunus* for the first time. *Phialophora bubakii* was combined in *Cadophora* and differentiated from *C. obscura*, which was resurrected. Asexual morphs of two *Proliferodiscus* species (Helotiales, Leotiomycetes) were described, including one new species, *Pr. ingens***sp. nov**. Two *Minutiella* species (Phaeomoniellales, Eurotiomycetes) were detected, including the new species *M. pruni-avium***sp. nov**. *Prunus avium* and *P. domestica* are reported as host plants of *Minutiella*.

## Introduction

In order to study the mycobiome of wood necroses of economically important *Prunus* species in Germany, a survey has been conducted using isolation techniques. Based on preliminary analyses of generated ITS sequences, several strains belonging to the Leotiomycetes and Eurotiomycetes were detected. Some of them were recently identified as species of *Collophorina* and related genera (Bien et al. 2020). Further strains showed morphological or genetical affiliation to the genera *Cadophora*, *Proliferodiscus* (Leotiomycetes) and *Minutiella* (Eurotiomycetes).

Leotiomycetes and Eurotiomycetes are both ecologically and morphologically highly diverse classes (Geiser et al. 2006, 2015; Wang et al. 2006b; LoBuglio and Pfister 2010; Johnston et al. 2019). Members of the Leotiomycetes have been described as plant pathogens, especially wood rot fungi, endophytes, nematode-trapping and mycorrhiza-forming fungi, as well as terrestrial and aquatic saprobes (Wang et al. 2006a; Hustad and Miller 2011). Eurotiomycetes are commonly known as saprotrophs and parasites of plants and animals; however, the number of pathogens is relatively low, compared to Sordariomycetes or Dothideomycetes (Geiser et al. 2006, 2015).

The genus *Cadophora* (Ploettnerulaceae, Helotiales, Leotiomycetes) was established in 1927 based on *C. fastigiata* (Lagerberg et al. 1927). Melin and Nannfeldt (1934) added five new species to *Cadophora*, while Davidson (1935) described two additional species that were isolated from stained wood or pulpwood products. Subsequently, Conant (1937) determined that *Phialophora* and *Cadophora* were congeneric and transferred the eight species of *Cadophora* to *Phialophora*. In the monograph of *Phialophora*, Schol-Schwarz (1970) included *C. fastigiata* and *C. malorum*, as well as *Margarinomyces bubakii* in *Phialophora*. Gams (2000) reinstated the genus *Cadophora* for phialophora-like species with more or less pigmented hyphae and pale phialides (*C. fastigiata*, *C. melinii*, *C. malorum*, *C. repens*). For some of these, a relationship with sexual morphs in some discomycete genera, such as *Mollisia* and *Pyrenopeziza*, has been demonstrated (Le Gal and Mangenot 1960, 1961). This connection was confirmed by LSU sequence analysis by Harrington and McNew (2003). However, to date, the type species of none of the genera has been epitypified. Currently, the genus *Cadophora* comprises 17 species. Species of *Cadophora* have been isolated from plants (e.g. Di Marco et al. 2004; Gramaje et al. 2011; Crous et al. 2015; Travadon et al. 2015; Walsh et al. 2018), soil (Kerry 1990; Hujslová et al. 2010; Agustí-Brisach et al. 2013) and decaying wood (Nilsson 1973; Blanchette et al. 2004, 2010).

Haines and Dumont (1983) compared specimens of *Dasyscyphus inspersus* (syn. *Peziza inspersa*) with the type species of the genera *Dasyscyphus* and *Lachnum* and revealed this species not to be congeneric with either of them. Based on spore, hair, paraphysis and subiculum morphology, they erected the new genus *Proliferodiscus* within the family Hyaloscyphaceae (Helotiales, Leotiomycetes). Today, the genus *Proliferodiscus* comprises eight species and is known from woody hosts in tropical and temperate regions worldwide (Haines and Dumont 1983; Spooner 1987; Cantrell and Hanlin 1997; McKenzie et al. 2000; Hofton et al. 2009; Han et al. 2014a; Haelewaters et al. 2018; Ekanayaka et al. 2019).

Crous and Gams (2000) described the genus *Phaeomoniella* (Celotheliaceae, Phaeomoniellales, Eurotiomycetes) based on *Pa. chlamydospora*, the causal agent of Esca disease of grapevine wood (Bertsch et al. 2013; Fontaine et al. 2016; Gramaje et al. 2018). Damm et al. (2010) discovered several new *Phaeomoniella* species from *Prunus* wood in South Africa that were combined in new genera by Crous et al. (2015). One of them, *Minutiella tardicola* (syn. *Pa. tardicola*), was characterised by the very slow growth of the cultures and minute pycnidia (Damm et al. 2010). Most members of Celotheliaceae have been found on *Prunus* (Damm et al. 2010) or other woody hosts from angiosperms and gymnosperms (Crous and Gams 2000; Nordén et al. 2005; Lee et al. 2006; Crous et al. 2008, 2009, 2015, 2016; Zhang et al. 2012; Úrbez-Torres et al. 2013; Yurkewich et al. 2017).

In this study, we aim to (1) systematically place strains isolated from necrotic wood of *Prunus* trees in Germany, as well as some additional strains tentatively identified as *Cadophora* within Leotiomycetes and Eurotiomycetes and (2) formally describe new taxa.

## Methods

### Sampling and fungal isolation

Branches with wood symptoms (e.g. canker, necroses, wood streaking, gummosis) were sampled from plum (*Prunus domestica*), sour cherry (*P. cerasus*) and sweet cherry (*P. avium*) orchards in Saxony, Lower Saxony and Baden-Württemberg, Germany, in 2015 and 2016. Additionally, a wood sample from a sour cherry tree located in a garden in Bavaria, as well as three strains previously isolated from wood of *P. salicina* in South Africa and two *Phialophora bubakii* strains, all tentatively identified as *Cadophora* spp. in preliminary analyses, were included. Wood pieces (5 × 5 × 5 mm) from the transition zone of symptomatic to non-symptomatic wood tissue, as well as pieces of the same size from non-symptomatic wood of the same branch, were surface sterilised (30 s in 70% ethanol, 1 min in 3.5% NaOCl, 30 s in 70% ethanol), washed for 1 min in sterilised water and placed on synthetic nutrient-poor agar medium (SNA; Nirenberg 1976), as well as oatmeal agar medium (OA; Crous et al. 2019), both supplemented with 100 mg/l penicillin, 50 mg/l streptomycin sulphate and 1 mg/l chloramphenicol. After incubation for several days at 25 °C, hyphal tips of developing fungi were transferred to SNA medium with a sterilised needle. Single-conidial isolates were obtained from the strains for further study. Sampling and isolation of the strains from South Africa was similar (Damm et al. 2007).

The strains are maintained in the culture collections of the Senckenberg Museum of Natural History Görlitz, Germany (GLMC), the Westerdijk Fungal Biodiversity Institute, Utrecht, The Netherlands (CBS) and the German Collection of Microorganisms and Cell Cultures, Braunschweig, Germany (DSMZ). Specimens (dried cultures), including type specimens were deposited in the fungarium of the Senckenberg Museum of Natural History Görlitz (GLMC).

### Morphological analysis

To enhance sporulation, autoclaved filter paper and double-autoclaved pine needles were placed on the surface of the SNA medium. The cultures were incubated in the dark at 25 °C. Colony growth and characters on SNA and OA, for some strains additionally on potato dextrose agar (PDA; Crous et al. 2019) and malt extract agar (MEA; Oxoid Ltd., England; 1.5% agar, Difco, USA), were noted after 2 and 4 wk. Colony colours were rated according to Rayner (1970). After 2 or 4 wk, microscopic preparations were made in clear lactic acid and observations and measurements (30 measurements per structure) were made with a Nikon SMZ18 stereomicroscope (SM) or with a Nikon Eclipse N*i*U light microscope with differential interference contrast (LM). Photographic images were captured with Nikon Digital Sight DSFi2 cameras installed on the above-mentioned microscopes, making use of the Nikon NIS-Elements software (v.4.30).

### DNA extraction, PCR amplification and sequencing

Of the forty-two strains isolated from *Prunus* wood in Germany, three strains from *Prunus* wood in South Africa, as well as two strains of *Phialophora bubakii* that were included in this study, 34, 4 and 8 strains had been identified as species of *Cadophora*, *Minutiella* and *Proliferodiscus*, respectively, in preliminary analyses based on ITS sequences. Twenty-two *Cadophora* strains, six *Proliferodiscus* strains, all *Minutiella* strains as well as an unidentified Leotiomycete strain were selected for phylogenetic analyses (Table 1).

**Table 1.** List of strains analysed in this study, with collection details and GenBank accession numbers.

Species	Accession no.^1^	Host/ substrate	Country	GenBank no.^2^			
				LSU	ITS	*TUB*	*EF1-α*
*Arboricolonus simplex*	GLMC 459^T^	*Prunus domestica*	Germany	**MN232924**	**MN232935**	–	–
*Cadophora africana*	CBS 120890^T^	*Prunus salicina*	South Africa	–	**MN232936**	**MN232967**	**MN232988**
*Cadophora bubakii* (as *Phialophora bubakii*)	CBS 198.30^T^	margarine	Czech Republic	–	MH855111	–	**MN232989**
*Cadophora luteo-olivacea*	GLMC 517	*Prunus domestica*	Germany	–	**MN232937**	**MN232968**	**MN233003**
	GLMC 1264	*Prunus domestica*	Germany	–	**MN232938**	**MN232969**	**MN233004**
	GLMC 1310	*Prunus domestica*	Germany	–	**MN232939**	**MN232970**	**MN233005**
	GLMC 1517	*Prunus domestica*	Germany	–	**MN232940**	**MN232971**	**MN233006**
	GLMC 1545	*Prunus domestica*	Germany	–	**MN232941**	**MN232972**	**MN233007**
*Cadophora novi-eboraci*	GLMC 239	*Prunus cerasus*	Germany	–	**MN232942**	**MN232973**	**MN232990**
	GLMC 273	*Prunus cerasus*	Germany	–	**MN232943**	**MN232974**	**MN232991**
	GLMC 274	*Prunus cerasus*	Germany	–	**MN232944**	**MN232975**	**MN232992**
	GLMC 342	*Prunus cerasus*	Germany	–	**MN232945**	**MN232976**	**MN232993**
	GLMC 688	*Prunus cerasus*	Germany	–	**MN232946**	**MN232977**	**MN232994**
	GLMC 1472	*Prunus cerasus*	Germany	–	**MN232947**	**MN232978**	**MN232995**
*Cadophora obscura* (as *Phialophora bubakii*)	CBS 269.33	fresh water	Sweden	–	**MN232948**	–	**MN232996**
*Cadophora prunicola*	CBS 120891^T^	*Prunus salicina*	South Africa	–	**MN232949**	**MN232979**	**MN232997**
	STEU 6103	*Prunus salicina*	South Africa	–	**MN232950**	–	–
	GLMC 276	*Prunus cerasus*	Germany	–	**MN232951**	**MN232980**	**MN232998**
	GLMC 362	*Prunus domestica*	Germany	–	**MN232952**	–	–
	GLMC 735	*Prunus cerasus*	Germany	–	**MN232953**	**MN232981**	**MN232999**
	GLMC 1574	*Prunus domestica*	Germany	–	**MN232954**	**MN232982**	**MN233000**
	GLMC 1633	*Prunus domestica*	Germany	–	**MN232955**	**MN232983**	**MN233001**
*Cadophora ramosa*	GLMC 377^T^	*Prunus cerasus*	Germany	–	**MN232956**	**MN232984**	**MN233002**
*Minutiella pruni-avium*	GLMC 1624^T^	*Prunus avium*	Germany	**MN232925**	**MN232957**	**MN232985**	–
	GLMC 1667	*Prunus avium*	Germany	**MN232926**	**MN232958**	**MN232986**	–
*Minutiella* sp.	GLMC 1636	*Prunus domestica*	Germany	**MN232927**	**MN232959**	–	–
	GLMC 1687	*Prunus domestica*	Germany	**MN232928**	**MN232960**	**MN232987**	–
*Proliferodiscus ingens*	GLMC 1751^T^	*Prunus avium*	Germany	**MN232929**	**MN232961**	–	–
*Proliferodiscus* sp.	GLMC 460	*Prunus domestica*	Germany	**MN232930**	**MN232962**	–	–
	GLMC 470	*Prunus domestica*	Germany	**MN232931**	**MN232963**	–	–
	GLMC 502	*Prunus domestica*	Germany	**MN232932**	**MN232964**	–	–
	GLMC 1301	*Prunus domestica*	Germany	**MN232933**	**MN232965**	–	–
	GLMC 1761	*Prunus avium*	Germany	**MN232934**	**MN232966**	–	–

^1^CBS: Culture collection of the Westerdijk Fungal Biodiversity Institute, Utrecht, The Netherlands; GLMC: Culture collection of Senckenberg Museum of Natural History Görlitz, Görlitz, Germany; STEU: University of Stellenbosch, Stellenbosch, South Africa. ^2^LSU: nuclear large subunit ribosomal DNA; ITS: internal transcribed spacers and intervening 5.8S nrDNA; *TUB*: β-tubulin gene;*EF1-α*: translation elongation factor 1-α gene. Sequences generated in this study are emphasised in bold face. ^T^ex-type cultures.

Genomic DNA of the isolates was extracted using the method of Damm et al. (2008). A partial sequence of the 28S nrDNA (LSU) and the 5.8S nuclear ribosomal gene with the two flanking internal transcribed spacers ITS1 and ITS2 (ITS) were amplified and sequenced using the primer pairs LR0R (Rehner and Samuels 1994) + LR5 (Vilgalys and Hester 1990) and ITS1F (Gardens and Bruns 1993) + ITS4 (White et al. 1990), respectively. Additionally, partial sequence of the βtubulin gene (*TUB*) and the translation elongation factor 1α (*EF-1α*) of strains belonging to the genus *Cadophora* were generated using the primer pairs BTCadF + BTCadR (Travadon et al. 2015) and EF1688F + EF11251R (Alves et al. 2008), respectively. The β-tubulin gene of the genus *Minutiella* was sequenced using the primer pair Bt2a + Bt2b (Glass and Donaldson 1995).

The PCR reaction mixture contained 1 μl of 1:10 DNA template, 2.5 μl 10X buffer (Peqlab, Erlangen, Germany), 1 μl of each primer (10mM), 2.5 μl MgCl_2_ (25mM), 0.1 μl *Taq* polymerase (0.5 U, Peqlab, Erlangen, Germany) and 2.5 μl of 2mM dNTPs. Each reaction was made up to a final volume of 20 μl with sterile water. DNA amplifications were carried out in a Mastercycler pro S (Eppendorf, Hamburg, Germany). The amplification conditions for ITS and *EF-1α* were: initial denaturation at 95 °C for 5 min; followed by 30 cycles of denaturation at 94 °C for 30 s, annealing at 51 °C for 30 s and extension at 72 °C for 60 s; and a final extension step at 72 °C for 3 min. The amplification conditions for the primer pair Bt2a + Bt2b were: initial denaturation at 94 °C for 4 min; followed by 38 cycles of denaturation at 94 °C for 60 s, annealing at 61 °C for 60 s and extension at 72 °C for 45 s; and a final extension step of 5 min at 72 °C. For amplifications of LSU and *TUB* with the primer pair BTCadF + BTCadR, the PCR conditions were set according to Paulin and Harrington (2000) and Travadon et al. (2015), respectively.

The PCR products were visualised on a 1% agarose gel and sequenced using the same primers by the Senckenberg Biodiversity and Climate Research Centre (BiK-F) laboratory (Frankfurt, Germany). The forward and reverse sequences were assembled by using BioEdit Sequence Alignment Editor (v. 7.2.5; Hall 1999).

### Phylogenetic analysis

For the phylogenetic analyses, sequences, especially those of ex-type strains, were downloaded from GenBank and added to the sequences generated in this study and those of the appropriate outgroup sequences in four datasets. In order to determine the generic placement of strain GLMC 459, sequences of close matches from blastn searches with its LSU and ITS sequences were combined with sequences of the phylogenetic reassessment of Hyaloscyphaceae by Han et al. (2014b) (dataset 1). Three datasets were generated to determine the systematic position of strains of the genera *Cadophora* (ITS, *TUB*, *EF-1α*; dataset 2), *Minutiella* (LSU, ITS, *TUB*; dataset 3) and *Proliferodiscus* (LSU, ITS; dataset 4). The datasets were aligned automatically using MAFFT v. 7.308 (Katoh et al. 2002, Katoh and Standley 2013) and manually adjusted where necessary.

The phylogenetical analyses were conducted using Bayesian Inference (BI), Maximum Likelihood (ML) and Maximum Parsimony (MP). For BI analyses, the best fit model of evolution for each partition was estimated by MEGA7 (Kumar et al. 2016). Posterior probabilities were determined by Markov Chain Monte Carlo sampling (MCMC) in MrBayes v. 3.2.6 (Huelsenbeck and Ronquist 2001; Ronquist and Huelsenbeck 2003) as implemented in Geneious v. 10.2.2 (Kearse et al. 2012), using the estimated models of evolution. For each dataset, four simultaneous Markov chains were run for 1 million generations and trees were sampled every 100^th^ generation. The first 2000 trees, which represent the burn-in phase of the analyses, were discarded and the remaining 8000 trees were used to calculate posterior probabilities in the majority rule consensus trees. The ML analyses were performed by RAxML v. 8.2.11 (Stamatakis 2006, 2014) as implemented in Geneious v. 10.2.2 (Kearse et al. 2012), using the GTRGAMMA model with the rapid bootstrapping and search for best scoring ML tree algorithm, including 1000 bootstrap replicates. The MP analyses were performed with MEGA7 (Kumar et al. 2016) using tree-bisection-reconnection (TBR) as the branch-swapping algorithm. The robustness of the trees was evaluated by 1000 bootstrap replicates and 10 random sequence additions. Tree length, consistency index, retention index and composite index of the resulting trees were calculated. The DNA sequences generated in this study were deposited in GenBank (Table 1), the alignments in TreeBASE (http://purl.org/phylo/treebase/phylows/study/TB2:S24703).

## Results

### Phylogenetic analyses

**Figure 1.**
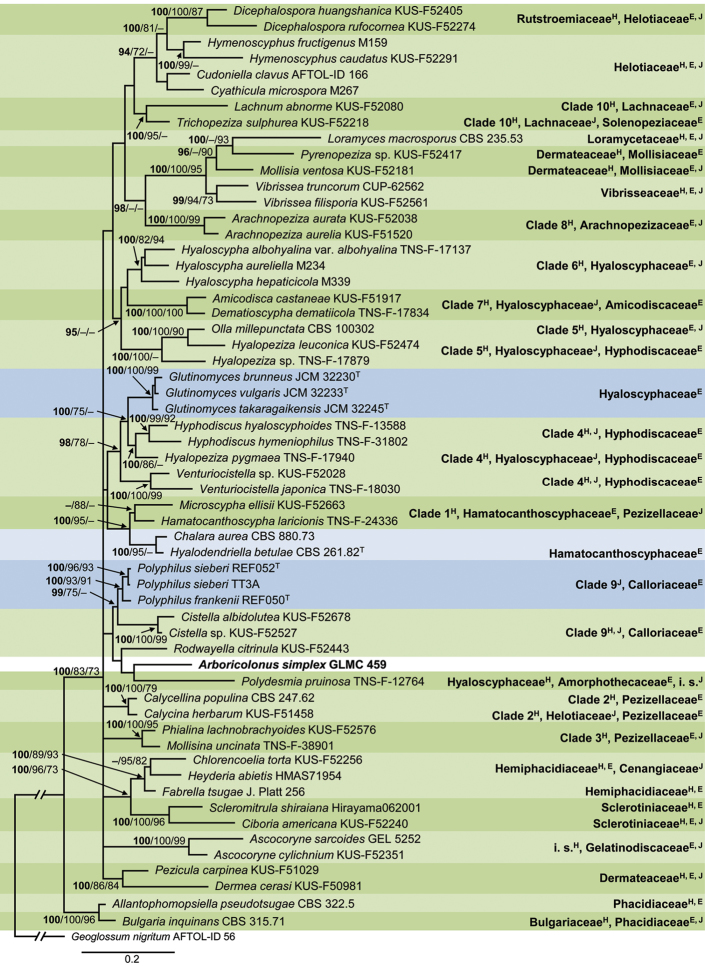
Phylogeny of dataset 1 obtained by Bayesian Inference analysis of the combined LSU and ITS sequence alignment for generic placement of strain GLMC 459. *Geoglossum nigritum* strain AFTOL-ID 56 is used as outgroup. BI posterior probability support values above 90% (bold) and ML and MP bootstrap support values above 70% are shown at the nodes. The strain, analysed in this study, is emphasised in bold. Green backgrounds indicate sequences included in the analyses of Han et al. (2014b). Blue backgrounds indicate close matches of GLMC 459 in blastn searches. Clades 1–10 of Hyaloscyphaceae, according to the analyses of Han et al. (2014b), are listed to the right. Family names are listed to the right, according to Han et al. (2014b, superscript H), Ekanayaka et al. (2019, superscript E) and Johnston et al. (2019, superscript J). Branches that are crossed by diagonal lines are shortened by 50%.

The combined sequence dataset 1 consisted of 59 isolates including the outgroup *Geoglossum nigritum* strain AFTOL-ID 56 and comprised 1540 characters, of which 436 characters were parsimony-informative, 578 variable and 885 constant. The gene boundaries on the LSU-ITS multi-locus alignment were as follows: LSU: 1–890 and ITS: 891–1540. The final ML optimisation likelihood of ML analysis was: lnL = -15669.074659. One most parsimonious tree was generated by MP analysis with tree length: 693 steps, consistency index: 0.298780, retention index: 0.555126 and composite index: 0.186644 and 0.165861 for all sites and parsimony informative sites, respectively. The BI phylogeny, including BI posterior probability values as well as ML and MP bootstrap support values, is shown in Fig. 1.

The clades in Fig. 1 represent all clades of the multi-locus phylogeny of the “Hyaloscyphaceae” by Han et al. (2014b) as well as clades formed by sequences of the closest matches from blastn searches with the ITS and LSU sequences of strain GLMC 459 in GenBank. Strain GLMC 459 from *P. domestica* in Germany forms a long single-strain clade that does not belong to any of the above-mentioned clades and is located close to *Polydesmia pruinosa* TNS-F-12764, strains belonging to Clade 9 in Han et al. (2014b) and a clade formed by three strains of *Polyphilus*. The clade, formed by GLMC 459 and these taxa, is not supported.

**Figure 2.**
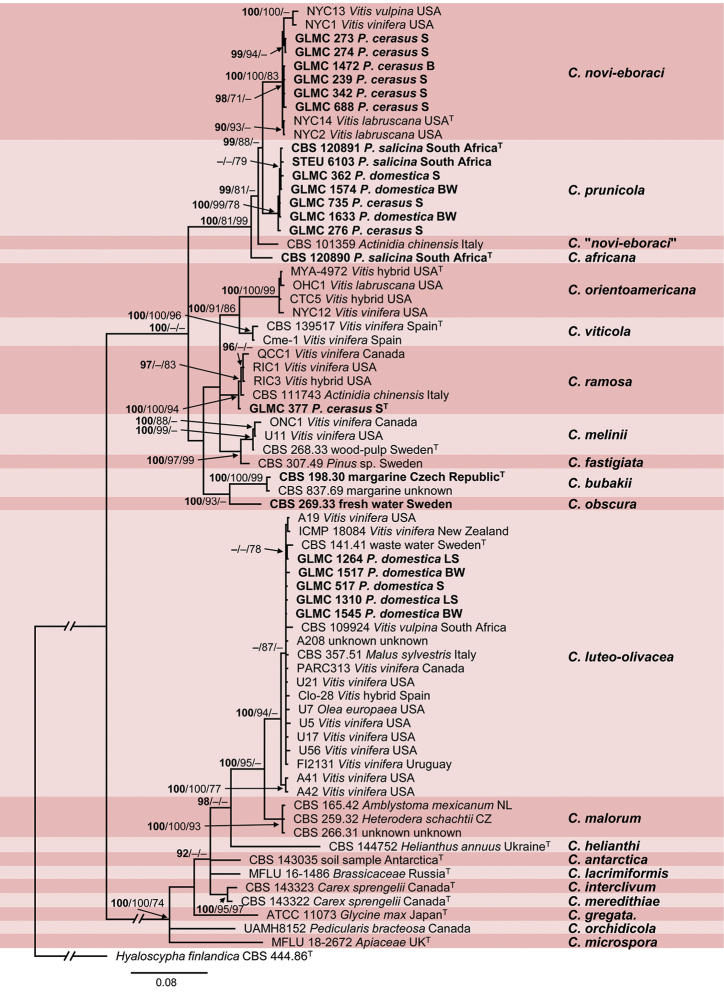
Phylogeny of dataset 2 obtained by Bayesian Inference analysis of the combined ITS, *TUB*, *EF-1α* sequence alignment of *Cadophora*. *Hyaloscypha finlandica* strain CBS 444.86 is used as outgroup. Host plant or substrate and country of isolation are given for every strain. For strains isolated from *Prunus* spp. in Germany, the German Federal State is given in abbreviation as location. Species names are listed to the right. BI posterior probability support values above 90% (bold), ML and MP bootstrap support values above 70% are shown at the nodes. The strains, analysed in this study, are emphasised in bold. Numbers of ex-type and ex-isotype strains are emphasised with a superscript T. Branches that are crossed by diagonal lines are shortened by 50%. B: Bavaria; BW: Baden-Württemberg; LS: Lower Saxony; S: Saxony.

The combined sequence dataset 2 of *Cadophora* consisted of 70 isolates including the outgroup *Hyaloscypha finlandica*CBS 444.86 and comprised 1594 characters, of which 498 characters were parsimony-informative, 692 variable and 859 constant. The gene boundaries in the multi-locus alignment were as follows: ITS: 1–575, *TUB*: 576–1133 and *EF-1α*: 1134–1594. Five most parsimonious trees were generated by MP analysis with tree length: 205 steps, consistency index: 0.536145, retention index: 0.931189 and composite index: 0.581425 and 0.499252 for all sites and parsimony informative sites, respectively. The BI phylogeny, including BI posterior probability values as well as ML (lnL = -9335.686864) and MP bootstrap support values, is shown in Fig. 2.

The phylogeny consists of two main clades belonging to 21 clades representing different *Cadophora* species. The two main clades are formed by BI and ML analyses; both are supported by BI (100%); however, only the second clade is supported by ML and MP analyses (100% and 74%, respectively). In the first main clade, six strains isolated from *P. cerasus* in Saxony and Bavaria form a well-supported clade (100/100/83% BI posterior probability, ML and MP bootstrap support values, respectively) with strains of *C. novi-eboraci* including its ex-type strain. A further five strains from *P. cerasus* and *P. domestica* in Saxony and Baden-Württemberg and two strains from *P. salicina* in South Africa form a well-supported clade (100/99/78%) that does not include any previously described species. One strain isolated from *P. salicina* in South Africa (CBS 120890) and a strain referred to as *C.* “*novi-eboraci*” (CBS 101359) form single-strain clades and belong to a well-supported clade with *C. novi-eboraci* and *C. prunicola* (100/81/99%). One strain isolated from *P. cerasus* in Saxony (GLMC 377) forms a well-supported clade (100/100/94%) with four strains referred to as *C.* “*spadicis*”. Within the second main clade, five strains isolated from *P. domestica* in all three sampling areas in Germany form a well-supported clade (100/94/–%) with 16 strains of *C. luteo-olivacea* including its ex-type strain. Two strains of *Phialophora bubakii*CBS 198.30 and CBS 837.69, both originating from margarine, form a well-supported clade (100/100/99%) sister to a third strain (CBS 269.33) from fresh water in Sweden that forms a single-strain clade. The clade formed by all three strains is well-supported (100/93/–%) as well.

**Figure 3.**
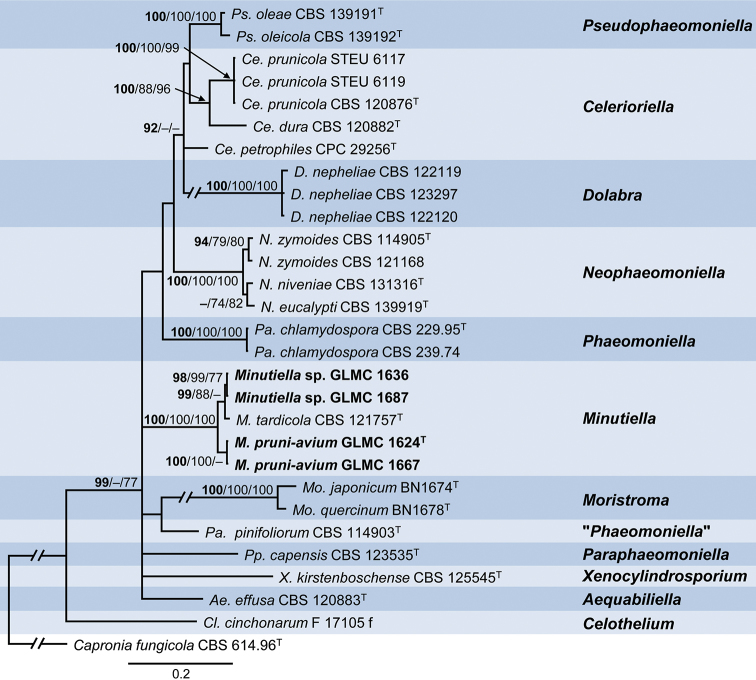
Phylogeny of dataset 3 obtained by Bayesian Inference analysis of the combined LSU, ITS, *TUB* sequence alignment of Phaeomoniellales, including *Minutiella*. *Capronia fungicola* strain CBS 614.96 is used as outgroup. BI posterior probability support values above 90% (bold), ML and MP bootstrap support values above 70% are shown at the nodes. The strains analysed in this study are emphasised in bold. Numbers of ex-type strains are emphasised with a superscript T. Branches that are crossed by diagonal lines are shortened by 50%.

The combined sequence dataset 3 consisted of 29 isolates of the Celotheliaceae and the outgroup *Capronia fungicola*CBS 614.96 and comprised 1904 characters, of which 486 characters were parsimony-informative, 685 variable and 1182 constant. The gene boundaries in the multi-locus alignment were as follows: LSU: 1–840 and ITS: 841–1482, *TUB*: 1483–1904. One most parsimonious tree was generated by MP analysis with tree length: 384 steps, consistency index: 0.558989, retention index: 0.779804 and composite index: 0.458467 and 0.435901 for all sites and parsimony informative sites, respectively. The BI phylogeny, including BI posterior probability values as well as ML (lnL = -9719.124620) and MP bootstrap support values, is shown in Fig. 3.

The 12 main clades of the phylogeny represent genera of the Celotheliaceae; all species for which sequences are available, are included. Four isolates from this study group in a well-supported clade (100/100/100%) with *Minutiella tardicola*. Two of the strains isolated from *P. domestica* form a well-supported sister clade (98/99/77 %) to the single-strain clade formed by the ex-type strain of *M. tardicola*. A further two strains isolated from *P. avium* form a well-supported clade (100/100/–%), sister to the clade consisting of *M. tardicola* and *Minutiella* sp.

**Figure 4.**
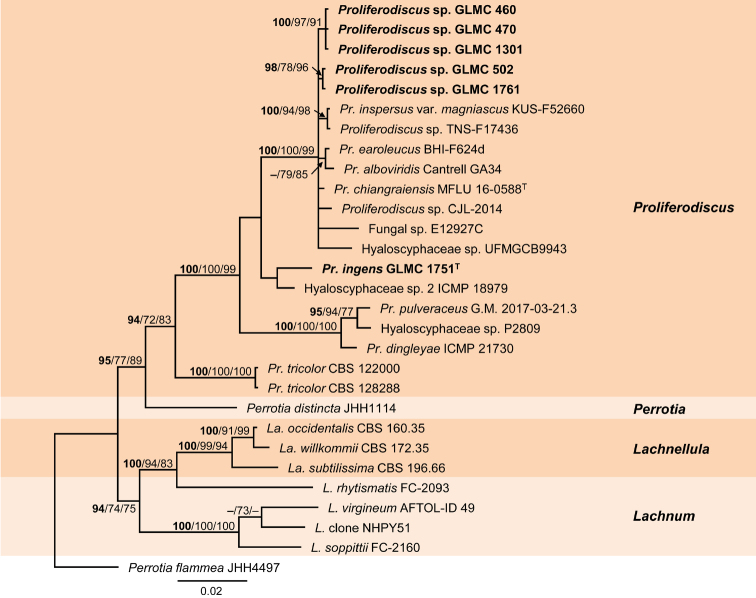
Phylogeny of dataset 4 obtained by Bayesian Inference analysis of the combined LSU, ITS sequence alignment of *Proliferodiscus* and close relatives. *Perrotia flammea* strain JHH4497 is used as outgroup. BI posterior probability support values above 90% (bold), ML and MP bootstrap support values above 70% are shown at the nodes. The strains, analysed in this study, are emphasised in bold. Numbers of ex-type strains are emphasised with a superscript T.

The combined sequence dataset 4 consisted of 29 isolates of *Proliferodiscus* and closely related genera including the outgroup *Perrotia flammea* JHH4497 and comprised 1385 characters, of which 152 characters were parsimony-informative, 204 variable and 1174 constant. The gene boundaries in the multi-locus alignment were as follows: LSU: 1–854 and ITS: 855–1385. Seven most parsimonious trees were generated by MP analysis with tree length: 263 steps, consistency index: 0.651341, retention index: 0.807611 and composite index: 0.526030 and 0.482422 for all sites and parsimony informative sites, respectively. The BI phylogeny obtained by Bayesian Inference, including BI posterior probability values as well as ML (lnL = -4019.800817) and MP bootstrap support values, is shown in Fig. 4.

The main clades represent closely related genera. Six strains from *Prunus* wood in Germany group in the *Proliferodiscus* clade. Five of them, from *P. avium* and *P. domestica*, cluster with seven ambiguously identified strains and the type strain of *Pr. chiangraiensis* in a well-supported clade (100/100/99%). Strain GLMC 1751 forms a single-strain clade sister to “Hyaloscyphaceae sp. 2” ICMP 18979.

### Taxonomy

Based on DNA sequence data and morphology, the 33 strains studied (Table 1) are assigned to four genera, of which seven species belong to *Cadophora*, two species to *Minutiella* and two species to *Proliferodiscus*, including 5 species that proved to be new to science and are described. Two strains, referred to as *Phialophora bubakii*, proved to belong to two distinct species within *Cadophora*. Strain GLMC 459 could not be assigned to any known genus and is therefore described as new genus. All species studied in culture are characterised below.

#### Arboricolonus

Taxon classificationFungiPhaeomoniellalesPhaeomoniellaceae

S.Bien & Damm gen. nov.

75121161-030A-5EFA-9B8D-B54874853688

832106

##### Type species.

*Arboricolonus simplex* S.Bien & Damm.

##### Etymology.

Referring to the life inside tree wood (*arbor* Lat. = tree + *colonus* = settler).

##### Description.

*Colonies* slow-growing, moist, white or buff colours on oatmeal agar medium, lacking aerial mycelium. *Sporulation* conidia formed on hyphal cells. *Conidiophores* reduced to conidiogenous cells. *Conidiogenous cells* enteroblastic, intercalary, reduced to short discrete phialides or, more often, collarettes formed directly on hyphal cells, collarettes short tubular to funnel-shaped. *Conidia* aggregated around the hyphae, small, hyaline, 1-celled, cylindrical, ovoidal to allantoid. *Vegetative hyphae and phialides* hyaline, smooth-walled, septate, branched.

#### Arboricolonus simplex

Taxon classificationFungiPhaeomoniellalesPhaeomoniellaceae

S.Bien & Damm sp. nov.

23BEFC07-66A4-5946-8E10-246CAFFDAFB0

832107

Figures 5A , 6

##### Type.

Germany, Saxony, orchard north of Wölkau, 50°58'42.3"N, 13°49'40.0"E, from brown wedge-shaped necrosis in wood of *Prunus domestica*, 16 Jan 2015, S. Bien leg., GLM-F106309 – ***holotype***; GLMC 459 = CBS 145520 = DSM 109147 – culture ex-type.

##### Etymology.

Named after the simple, reduced conidiophores.

**Figure 5.**
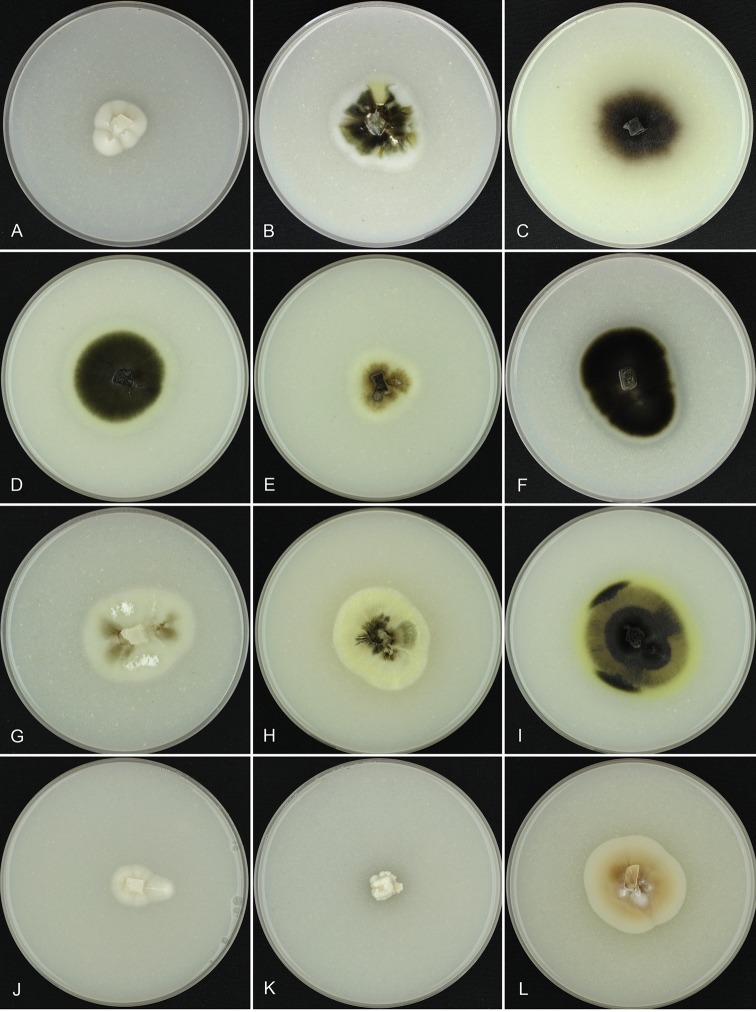
Colony surface of analysed strains on OA medium. **A***Arboricolonus simplex*GLMC 459^T^**B***Cadophora africana*CBS 120890^T^**C***C. bubakii*CBS 198.30^T^**D***C. luteo-olivacea*GLMC 1264 **E***C. novi-eboraci*GLMC 1472 **F***C. obscura*CBS 269.33 **G***C. prunicola*CBS 120891^T^**H***C. prunicola*GLMC 1633 **I***C. ramosa*GLMC 377^T^**J***Minutiella pruni-avium*GLMC 1624^T^**K***Proliferodiscus ingens*GLMC 1751^T^**L***Proliferodiscus* sp. GLMC 460. Cultures **A, J–L** after 4 wk. Cultures **B–I** after 2 wk. Strains with a superscript T are ex-type cultures.

**Figure 6.**
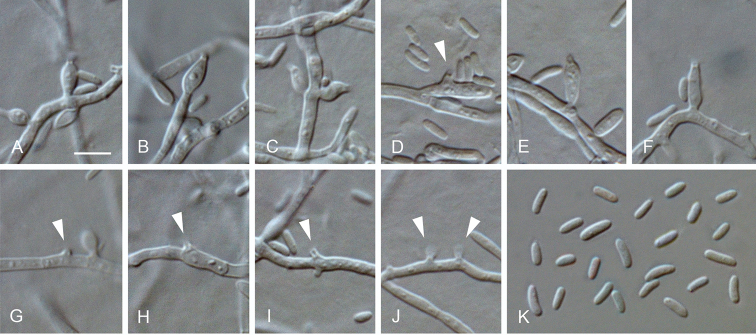
*Arboricolonus simplex* gen. et sp. nov. **A–J** conidiogenous cells (arrows indicate conidiogenous openings or short necks) **K** conidia **A–K** from SNA**A–K**LM. Scale bar: 5 μm (**A** applies to **B–K**).

##### Description.

*Sexual morph* not observed. *Asexual morph on SNA. Vegetative mycelium* hyaline, smooth-walled, septate, branched, 1–3 µm wide, sometimes hyphal cells inflated and constricted at the septa, chlamydospores absent. *Sporulation* abundant, conidia formed on hyphal cells. *Conidiophores* reduced to conidiogenous cells. *Conidiogenous cells* enteroblastic, hyaline, smooth-walled, integrated or terminal, discrete phialides, ampulliform to navicular, 4–7 × 2–3 μm, often reduced to small necks or openings on hyphae, opening 0.5–1 µm wide, collarettes short tubular to funnel-shaped, 0.5–1 µm long, periclinal thickening sometimes visible. *Conidia* aggregated in heads or slimy masses around hyphae, hyaline, smooth-walled, aseptate, straight to ± curved, cylindrical, elongate ovoidal to allantoid, with one end rounded, the other end rounded to truncate, 3–4(–4.5) × 1–1.5(–2) µm, mean ± SD = 3.6 ± 0.6 × 1.3 ± 0.2 µm, L/W ratio = 2.8.

##### Culture characteristics.

*Colonies on OA* flat to slightly raised with an entire to undulate margin, hyaline, whitish to buff, lacking aerial mycelium, reverse same colours, 2–4 mm diam. in 2 wk, 6–10 mm diam. in 4 wk; *on SNA* flat to slightly raised with an entire to undulate margin, hyaline to whitish, lacking aerial mycelium, reverse same colours, 1–2 mm diam. in 2 wk, 3–6 mm diam. in 4 wk.

##### Notes.

The morphology of *Arboricolonus simplex* is reminiscent of collophorina-like species regarding the colonies that are slow growing, the lack of aerial mycelium and the conidiogenous cells that are mostly reduced to short necks or openings with collarettes on hyphae (Damm et al. 2010; Bien et al. 2020). In contrast to these genera, microcyclic conidiation has not been observed in *Arboricolonus*. This genus belongs to the Leotiomycetes as well; however, it is not closely related to *Collophorina* and collophorina-like genera (Phacidiales) treated by Bien et al. (2020). A class-wide phylogenetic analysis of LSU-ITS places it within the order Helotiales (data not shown).

A blastn search with the ITS sequence of *A. simplex* in GenBank resulted in uncultured and unidentified strains with ≤ 92% identity, for example, an uncultured Helotiales clone from soil in the USA (HQ021771, JH Vineis et al., unpubl. data), while the closest matches with strains, identified at least to the genus level, were strains of *Glutinomyces vulgaris* with 90% identity (e.g. LC218288; Nakamura et al. 2018). The closest matches in a blastn search with the LSU sequence were, with ≤ 97% identity, the ex-type strain of *Hyalodendriella betulae* (EU040232; Crous et al. 2007), a strain identified as *Chalara aurea* (MH872551; Vu et al. 2019) and strains belonging to *Polyphilus sieberi* (e.g. MG719708; Ashrafi et al. 2018).

#### Cadophora africana

Taxon classificationFungiChaetothyrialesHerpotrichiellaceae

Damm & S.Bien sp. nov.

08CD29D5-E482-5CA3-A9D0-69069B215F8A

832108

Figures 5B , 7

##### Type.

South Africa, Western Cape Province, Franschhoek, from necrosis in wood of *Prunus salicina* close to old pruning wound, 10 June 2004, U. Damm leg., CBS H-19984 – ***holotype***; GLM-F117479 – ***isotype***; CBS 120890 = STE-U 6203 = GLMC 1892 – culture ex-type.

##### Etymology.

Named after the continent of origin, Africa.

**Figure 7.**
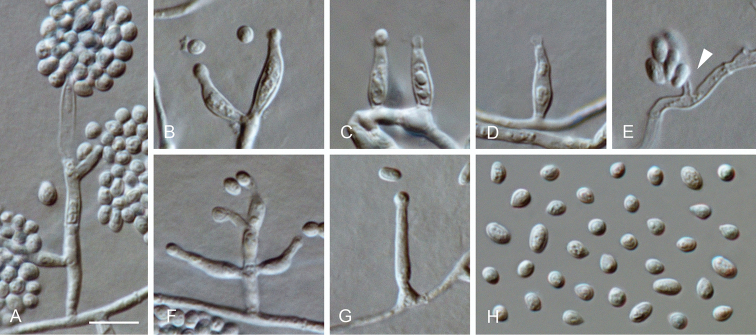
*Cadophora africana* sp. nov. **A–G** conidiophores and conidiogenous cells (arrow indicates a short neck) **H** conidia **A–H** from SNA**A–H**LM. Scale bar: 5 μm (**A** applies to **B–H**).

##### Description.

*Sexual morph* not observed. *Asexual morph on SNA. Vegetative mycelium* hyaline, smooth-walled, septate, branched, 1–3 µm wide, hyphal cells sometimes inflated and constricted at the septa, sometimes becoming brown with age, chlamydospores absent. *Sporulation* abundant, conidia formed on hyphal cells. *Conidiophores* hyaline, smooth-walled, mesotonously branched, occasionally with acropleurogenous branching, up to 35 µm long. *Conidiogenous cells* enteroblastic, hyaline, smooth-walled, discrete conidiogenous cells cylindrical to navicular, often constricted and sometimes widened at the base, 8–18 × 1.5–3 µm, necks cylindrical, 1–2 × 1–1.5 µm, collarettes distinct, cylindrical to narrowly funnel-shaped, 0.5–1.5 µm long, 1–1.5 µm wide at the upper edge, opening 1–1.5 µm wide, periclinal thickening observed. *Conidia* aggregated in heads, hyaline, smooth-walled, aseptate, mostly globose to subglobose or obovoid to tear-shaped, sometimes ellipsoidal, (2–)2.5–4(–4.5) × (1.5–)2–2.5(–3) µm, mean ± SD = 3 ± 0.5 × 2.1 ± 0.2 µm, L/W ratio = 1.4.

##### Culture characteristics.

*Colonies on SNA* flat with an entire to undulate margin, white to buff, sometimes grey olivaceous to olivaceous, lacking aerial mycelium, reverse same colours, 6–14 mm diam. in 2 wk (25 °C in the dark); *Colonies on OA* flat with an entire to undulate margin, primrose to amber, grey olivaceous to olivaceous black, often with a white margin, partly covered by floccose white aerial mycelium, reverse buff to grey olivaceous, 22–30 mm diam. in 2 wk (25 °C in the dark); *Colonies on PDA* flat to raised, entire edge, short aerial mycelium, pale buff, after > 2 wk with pale olivaceous to pale olivaceous grey patches or sectors, reverse same colours, 30 mm diam. in 2 wk (20 °C). *Colonies on MEA* flat to low umbonate, with entire edge, abundant velvety aerial mycelium, mycelium and surface white to very pale smoke-grey; reverse very pale luteous, ochreous to buff, in diffuse daylight with concentric oliveceous-grey rings, 30 mm diam. in 2 wk (20 °C).

##### Notes.

*Cadophora africana* was isolated once from *P. salicina* in South Africa. *Cadophora africana*, as well as *C. bubakii* and *C. ramosa*, form subglobose conidia. However, conidia of *C. africana* are mostly globose to subglobose, sometimes even tear-shaped, while those of *C. ramosa* are often ellipsoidal, elongate-ellipsoidal to cylindrical and the portion of subglobose conidia in *C. bubakii* is comparatively low. Therefore, conidia of both species are on average longer (4.9 µm and 3.6 µm, respectively) than those of *C. africana* (3 µm) and with a larger L/W ratio (2.2 and 2.1, respectively; *C. africana*: 1.4).

The ITS sequence of *C. africana* strain CBS 120890 differs in eleven nucleotides from the ex-type strain of *C. prunicola* and in nine nucleotides, both from the ex-type strain of *C. novi-eboraci* NYC14 and from strain CBS 101359. The differences to these strains exceed 30 and 18 nucleotides in the *TUB* and *EF-1α* sequences, respectively. The closest match in a blastn search with the ITS sequence of *C. africana* is strain NYC13 of *C. novi-eboraci* (identity 98.48%), which is included in our phylogeny.

#### Cadophora bubakii

Taxon classificationFungiChaetothyrialesHerpotrichiellaceae

(Laxa) Damm & S.Bien comb. nov.

4C0CEF39-EADC-5AD1-AE20-7E90E13E6E69

Figures 5C , 8

Margarinomyces bubakii Laxa, Zentbl. Bakt. ParasitKde, Abt. II 81: 392. 1930. (Basionym)
≡ Phialophora bubakii (Laxa) Schol-Schwarz, Persoonia 6 (1): 66. 1970.

##### Type.

Czech Republic, Prague, from a margarine factory, margarine, O. Laxa leg., collection date unknown (isolated by O. Laxa, deposited in CBS collection by O. Laxa probably 1930), CBS H-491, CBS H-7316, GLM-F117482 – ***isotypes***; CBS 198.30 = IMI 24000 = NCTC 3273 = VKM F-162 = LM 288 = LM 793 = GLMC 1895 – culture ex-isotype.

**Figure 8.**
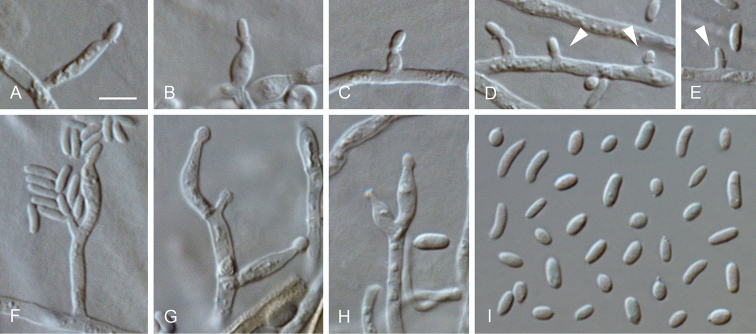
*Cadophora bubakii* comb. nov. **A–H** conidiophores and conidiogenous cells (arrows indicate short necks) **I** conidia **A–I** from SNA**A–I**LM. Scale bar: 5 μm (**A** applies to **H–I**).

##### Description.

*Sexual morph* not observed. *Asexual morph on SNA. Vegetative mycelium* hyaline, smooth-walled, septate, branched, 1–3 µm wide, sometimes becoming brown with age, chlamydospores absent. *Sporulation* abundant, conidia formed on hyphal cells. *Conidiophores* hyaline, smooth-walled, occasionally with acropleurogenous branching, up to 26 µm long. *Conidiogenous cells* enteroblastic, hyaline, smooth-walled, discrete conidiogenous cells cylindrical to navicular, often slightly inflated having a flask-shaped appearance, often constricted at the base, 3–12 × 1.5–3.5 µm, necks cylindrical, 1–2.5 × 1–2 µm, collarettes distinct, cylindrical to funnel-shaped, 0.5–1 µm long, 1–1.5 µm wide at the upper edge, opening 1–1.5 µm wide, periclinal thickening observed. *Conidia* aggregated in heads, hyaline, smooth-walled, aseptate, subglobose to ellipsoidal or cylindrical with both ends rounded, straight or slightly curved, (2–)2.5–4.5(–6) × 1.5–2 µm, mean ± SD = 3.6 ± 0.9 × 1.7 ± 0.2 µm, L/W ratio = 2.1.

##### Culture characteristics.

*Colonies on SNA* flat with an entire to undulate margin, white, lacking aerial mycelium, reverse same colour, 36–56 mm diam. in 2 wk (25 °C in the dark); *Colonies on OA* flat with an entire to undulate margin, olivaceous to olivaceous black, sometimes covered by floccose aerial mycelium, olivaceous grey, reverse same colours, 24–27 mm diam. in 2 wk (25 °C in the dark).

##### Notes.

The genus *Margarinomyces* was described 1930 with *Ma. bubakii* as type species after causing problems in a margarine factory in Czech Republic by forming greenish-black spots on and in margarine cubes that smelled like bitter-almond (benzaldehyde) (Laxa 1930). The fungus was shown to survive 20 min at 60 °C and to be resistant to organic preservatives such as sodium benzoate that was, however, only tolerated in margarine up to a concentration of 0.2% (Laxa 1930). According to the CBS website, strain CBS 198.30 is ex-isotype of *Ma. bubakii*. Schol-Schwarz (1970) included *Ma. bubakii* in *Phialophora* and considered *C. obscura* as a synonym. The genus *Margarinomyces* had been included in *Phialophora* by Gams and McGinnis (1983), though excluded by Cole and Kendrick (1973), but *Ma. bubakii* has never been considered as a species of *Cadophora* before. All nine *Margarinomyces* species had been combined in other genera, most of them in *Coniochaeta* (http://www.indexfungorum.org).

*Cadophora bubakii* (strain CBS 198.30) differs from *C. obscura* (strain CBS 269.33) by forming conidiogenous cells that are often slightly inflated and therefore flask-shaped, while those of CBS 269.33 are mostly narrow cylindrical. Conidia of strain CBS 198.30 are sometimes subglobose and, on average, distinctly shorter than the ellipsoidal to cylindrical conidia of CBS 269.33. Moreover, colonies of CBS 198.30 grow faster. Van Beyma (1943) compared *Ma. bubakii* and *Ph. obscura* and mentioned flask-shaped conidiogenous cells and a faster colony growth rate of *Ma. bubakii* and narrow phialides of *Ph. obscura* as well. However, the conidia shape of both species was described and illustrated as rod-shaped.

The ITS sequences of the two *C. bubakii* strains included in the phylogeny of this study, CBS 198.30 and CBS 837.69, are identical but differ both in 19 nucleotides from that of the *C. obscura* strain CBS 269.33. The *EF-1α* sequence of the two species differs in 31 nucleotides. The *TUB* sequences of CBS 198.30 and CBS 269.33 were not able to be aligned with each other and the rest of the dataset and therefore excluded from the phylogeny.

A blastn search with the ITS sequence of CBS 198.30 resulted in high similarities (99.82% and 99.64%) with “*Ph. bubakii*” strains CBS 837.69 (included in our analysis) and CBS 836.69, both isolated from margarine, as well as CBS 834.69, isolated from wood pulp of *Populus tremula* (Vu et al. 2019).

#### Cadophora luteo-olivacea

Taxon classificationFungiChaetothyrialesHerpotrichiellaceae

(J.F.H.Beyma) T.C.Harr. & McNew

E0F214F6-A948-5ED1-B5B3-03CE8FB164C9

Figures 5D , 9

##### Description.

*Sexual morph* not observed. *Asexual morph on SNA. Vegetative mycelium* hyaline, smooth-walled, septate, branched, 1–10 µm wide, hyphal cells often, sometimes very strongly inflated and constricted at the septa, chlamydospores absent. *Sporulation* abundant, conidia formed on hyphal cells. *Conidiophores* hyaline, smooth-walled, simple or septate and branched, up to 40 µm long. *Conidiogenous cells* enteroblastic, hyaline, smooth-walled, cylindrical to ± inflated, 3–14 × 1.5–4 µm, sometimes integrated, necks cylindrical, 0.5–3 µm long, collarettes funnel-shaped, 1–1.5 µm long, 1–2 µm wide at the upper edge, opening 1–1.5 µm wide, periclinal thickening not observed. *Conidia* aggregated in heads, hyaline, smooth-walled, aseptate, cylindrical, rarely ellipsoidal, straight, sometimes slightly curved, both ends rounded, conidia of strain GLMC 1310 measure (2–)4–7(–8) × 1.5–2.5 µm, mean ± SD = 5.3 ± 1.4 × 2.0 ± 0.3 µm, L/W ratio = 2.7, while those of GLMC 1264 are longer, measuring (3–)5–8(–10) × 1.5–2 µm, mean ± SD = 6.4 ± 1.6 × 1.8 ± 0.2 µm, L/W ratio = 3.5.

**Figure 9.**
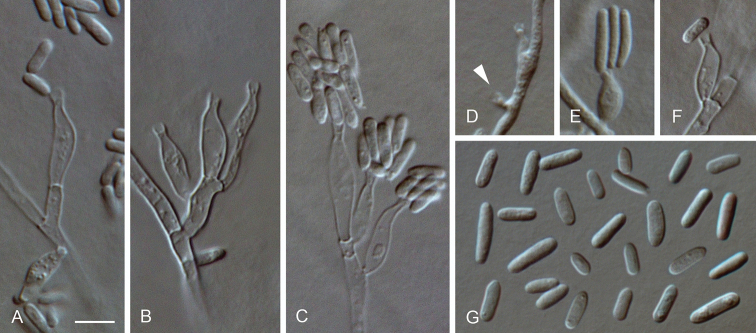
*Cadophora luteo-olivacea*. **A–F** conidiophores and conidiogenous cells (arrow indicates a short neck) **G** conidia **A–G** from SNA**A–G**LM. Scale bar: 5 μm (**A** applies to **B–G**).

##### Culture characteristics.

*Colonies on SNA* flat with an entire margin, hyaline, sometimes filter paper partly pale olivaceous to olivaceous, lacking aerial mycelium, reverse same colours, strains GLMC 1264 and GLMC 1310 5–15 mm diam., strains GLMC 517 and GLMC 1501 32–43 mm diam. in 2 wk (25 °C in the dark); *Colonies on OA* flat with an entire margin, buff, olivaceous buff, olivaceous to olivaceous black, lacking aerial mycelium or partly covered by pale grey aerial mycelium, reverse same colours, 28–44 mm diam. in 2 wk (25 °C in the dark).

##### Notes.

In total, 12 strains of *C. luteo-olivacea* were isolated from *Prunus domestica* in Baden-Württemberg (3), Lower Saxony (8) and Saxony (1). Two strains from Baden-Württemberg, two strains from Lower Saxony and the strain from Saxony had been selected for the phylogenetic analyses. The complete sequence dataset of *C. luteo-olivacea*, including reference strains, exhibits a variation of up to five nucleotides within ITS, up to nine nucleotides within *TUB* and up to 16 nucleotides within *EF-1α* sequences. The ITS sequences of the strains from this study are identical with those of the ex-type strain, except for GLMC 1517, which differs in five nucleotides, while all *TUB* sequences of our isolates differ in eight to nine nucleotides from the ex-type strain. The *EF-1α* sequence of all strains from this study differs in five nucleotides from the ex-type strain, except for GLMC 1264 with no differences.

##### Material examined.

Germany, Lower Saxony, Hollern-Twielenfleth, orchard, 53°36'13.6"N, 9°31'50.8"E, from brown wedge-shaped necrosis in wood of *Prunus domestica*, 8 Oct. 2015, S. Bien leg., GLM-F107114, culture GLMC 1264 = CBS 145524 = DSM 109143; Lower Saxony, Hollern-Twielenfleth, orchard, 53°36'13.6"N, 9°31'50.8"E, from brown wedge-shaped necrosis in wood of *P. domestica*, 8 Oct 2015, S. Bien leg., GLM-F107160, culture GLMC 1310 = CBS 145525 = DSM 109142; Saxony, in orchard north of Wölkau, 50°58'42.3"N, 13°49'40.0"E, from brown wedge-shaped necrosis in wood of *P. domestica*, 16 Jan 2015, S. Bien leg., GLM-F106367, culture GLMC 517; Baden-Württemberg, orchard west of Nussbach, 48°31'55.8"N, 8°00'52.4"E, from brown wedge-shaped necrosis in wood of *P. domestica*, 23 Aug 2016, S. Bien leg., GLM-F110581, culture GLMC 1501; Baden-Württemberg, orchard east of Nussbach, 48°31'57.3"N, 8°01'49.6"E, from brown wedge-shaped necrosis in wood of *P. domestica*, 23 Aug 2016, S. Bien leg., GLM-F110597, culture GLMC 1517 = CBS 145526 = DSM 109141.

#### Cadophora novi-eboraci

Taxon classificationFungiChaetothyrialesHerpotrichiellaceae

Travadon, D.P.Lawr., Roon.-Lath., Gubler, W.F.Wilcox, Rolsh. & K.Baumgartner

77C2EB15-2DA5-519A-9755-0B3672A3B3A6

Figures 5E , 10

##### Description.

*Sexual morph* not observed. *Asexual morph on SNA. Vegetative mycelium* hyaline, smooth-walled, septate, branched, 1–4 µm wide, sometimes hyphae inflated and constricted at the septa, chlamydospores absent. *Sporulation* abundant, conidia formed on hyphal cells. *Conidiophores* hyaline, smooth-walled, mostly simple, rarely septate and branched, up to 20 µm. *Conidiogenous cells* enteroblastic, hyaline, smooth-walled, often integrated, discrete conidiogenous cells ampulliform, ellongate-ampulliform to navicular, 7–17 × 1.5–3 µm, necks cylindrical, 1–1.5 × 1.5–5.5 µm, collarettes cylindrical to narrowly funnel-shaped, 1.5–2 µm long, 0.5–1.5 µm wide at the upper edge, opening 0.5–1 µm, periclinal thickening sometimes observed. *Conidia* aggregated in heads, hyaline, smooth-walled, aseptate, cylindrical, elongate-ellipsoidal to ellipsoidal, straight, rarely slightly curved, with both ends rounded, (3–)4.5–6.5(–8.5) × 1.5–2(–2.5) µm, mean ± SD = 5.4 ± 1.1 × 1.8 ± 0.4 µm, L/W ratio = 2.9.

**Figure 10.**
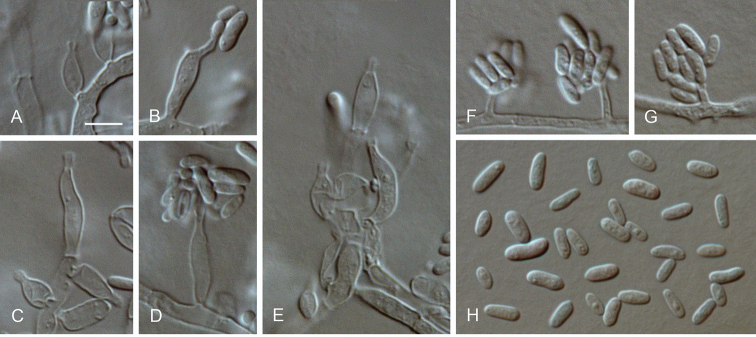
*Cadophora novi-eboraci***A–G** conidiophores and conidiogenous **H** conidia **A–H** from SNA**A–H**LM. Scale bar: 5 μm (**A** applies to **B–H**).

##### Culture characteristics.

*Colonies on SNA* flat with an entire to undulate margin, hyaline to pale smoke grey, filter paper partly pale luteous to very pale smoke grey, lacking aerial mycelium, reverse same colours, 5–7 mm diam. in 2 wk (25 °C in the dark); *Colonies on OA* flat with an entire to undulate margin, fawn to umber with a pale luteous to luteous margin, partly covered by floccose white aerial mycelium, reverse fawn, pale olivaceous to pale luteous, 18 mm diam. in 2 wk (25 °C in the dark).

##### Notes.

In total, eight strains of *C. novi-eboraci* were isolated from *Prunus cerasus* in Saxony (7) and Bavaria (1). Five of the strains from Saxony and the strain from Bavaria had been selected for the phylogenetic analyses. The complete sequence dataset of *C. novi-eboraci* exhibits a certain amount of variation in the loci analysed. The ITS and *EF-1α* sequences exhibited a maximum of one and two nucleotide differences to those of the ex-type strain NYC14, respectively. The *TUB* sequences were more variable; the *TUB* sequence of strain NYC13 differs in 15 nucleotides from that of NYC14. The *TUB* sequences of the strains from this study only differ with a maximum of two nucleotides from the ex-type strain.

##### Material examined.

Germany, Bavaria, in garden east of Wolferszell, 48°57'38.8"N, 12°38'24.9"E, from non-symptomatic wood of *Prunus cerasus*, 2 Oct 2016, J. Simmel leg., GLM-F110552, culture GLMC 1472 = CBS 145758 = DSM 109145.

#### Cadophora obscura

Taxon classificationFungiChaetothyrialesHerpotrichiellaceae

Nannf., Svenska Skogsvårdsföreningens Tidskrift 50: 418 (1934)

201F384E-596F-5348-B571-28F4EB810690

Figures 5F , 11

≡ Phialophora obscura (Nannf.) Conant, Mycologia 29(5): 598 (1937)

##### Type.

Sweden, Umeå, Sofiehem, Sofiehems trämassefabrik, from fresh water, E Melin leg., collection date unknown, UPS F-153532 – ***holotype*** (not seen); unknown source, E Melin, collection date unknown (isolated by E Melin and JA Nannfeldt No. 389:11, deposited in CBS collection by E Melin probably 1933), CBS H-7589, CBS H-7590, GLM-F117483 – ***isotypes***; CBS 269.33 = GLMC 1896 – culture ex-isotype.

**Figure 11.**
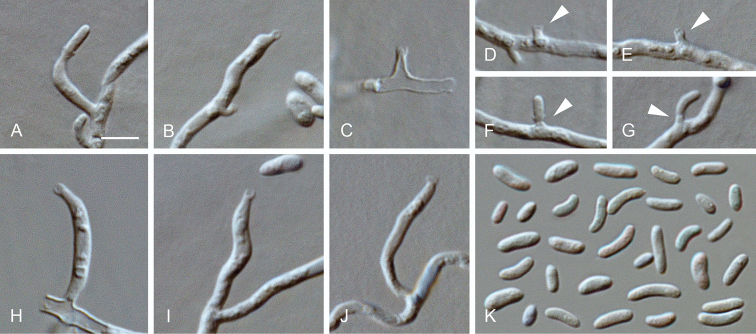
*Cadophora obscura***A–J** conidiophores and conidiogenous cells (arrows indicate short necks) **K** conidia **A–K** from SNA**A–K**LM. Scale bar: 5 μm (**A** applies to **B–K**).

##### Description.

*Sexual morph* not observed. *Asexual morph on SNA. Vegetative mycelium* hyaline, smooth-walled, septate, branched, 1–3.5 µm wide, sometimes becoming brown with age, chlamydospores absent. *Sporulation* abundant, conidia formed on hyphal cells. *Conidiophores* reduced to conidiogenous cells. *Conidiogenous cells* enteroblastic, hyaline, smooth-walled, discrete conidiogenous cells cylindrical to navicular, often bent, sometimes constricted at the base, 3–19 × 2–3 µm, necks cylindrical, 1–3.5 × 1.5–2 µm, collarettes distinct, cylindrical to funnel-shaped, 0.5–1.5 µm long, 1–1.5 µm wide at the upper edge, opening 1–1.5 µm wide, periclinal thickening observed. *Conidia* aggregated in heads, hyaline, smooth-walled, aseptate, ellipsoidal to cylindrical, mostly slightly curved, with both ends rounded, (3–)3.5–6(–7) × 1.5–2(–2.5) µm, mean ± SD = 4.8 ± 1.2 × 1.7 ± 0.3 µm, L/W ratio = 2.8.

##### Culture characteristics.

*Colonies on SNA* flat with an entire to fimbriate margin, white to cinnamon, filter paper buff to olivaceous, lacking aerial mycelium, reverse same colours, 14–16 mm diam. in 2 wk (25 °C in the dark); *Colonies on OA* flat with an entire margin, olivaceous black to greenish-black, with honey to white margin, sometimes covered by floccose, olivaceous grey aerial mycelium, reverse same colours, 14–20 mm diam. in 2 wk (25 °C in the dark).

##### Notes.

*Cadophora obscura* was originally described by Melin and Nannfeldt (1934) from freshwater in Sweden. According to the CBS website, strain CBS 269.33 is an “ex-isotype” culture. However, as Melin and Nannfeldt (1934) only isolated this species once and stated that they handed the strains from their study over to the Centraalbureau voor Schimmelcultures in Baarn now Westerdijk Fungal Biodiversity Institute, this can only be the ex-holotype strain. However, we were not able to allocate this strain to the holotype without doubt.

This species had previously been regarded as belonging to the genus *Phialophora* (Medlar 1915) and as a synonym of *Phialophora bubakii* (Schol-Schwarz 1970). However, based on the phylogeny of this study, both species are distinct species of the genus *Cadophora*. *Cadophora obscura* (CBS 269.33) differs from *C. bubakii* (CBS 198.30) by forming conidiogenous cells that are mostly narrow cylindrical, while those of CBS 198.30 are often flask-shaped. Conidia of *C. obscura* are distinctly longer than those of *C. bubakii*; subglobose-shaped conidia were not observed. Colony growth is slower compared to *C. bubakii*.

The ITS and *EF-1α* sequences of the ex-type strains of *C. bubakii* and *C. obscura* differ in 19 and 31 nucleotides, respectively. The *TUB* sequences of the two species were excluded from the analyses (see Notes of *C. bubakii*).

The ITS sequence of CBS 269.33 is 100% identical with three strains isolated from archaeological wood in Greenland (586-C, 592-B, 588-A, NB Pedersen et al., unpubl. data).

#### Cadophora prunicola

Taxon classificationFungiChaetothyrialesHerpotrichiellaceae

Damm & S.Bien sp. nov.

EAFA16A9-588B-5AB6-A3DF-8D83DC562E26

832109

Figures 5G, H , 12

##### Type.

South Africa, Western Cape province, Franschhoek, from reddish-brown necrosis in wood of *Prunus salicina* close to an old pruning wound, 10 June 2004, U. Damm leg., CBS H-19985 – ***holotype***; GLM-F117487 – ***isotype***; CBS 120891 = STE-U 6202 = GLMC 1902 – culture ex-type.

##### Etymology.

Named after its host genus, *Prunus* + suffix -cola (dweller).

**Figure 12.**
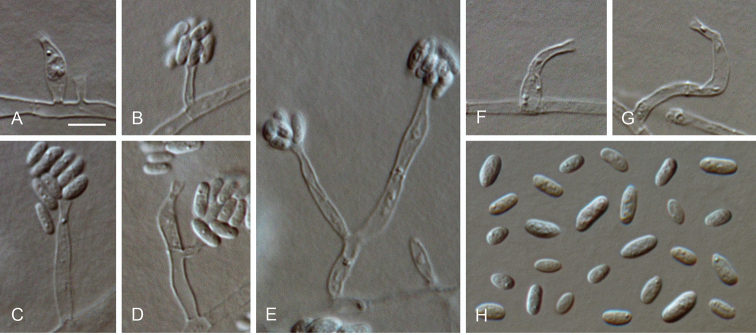
*Cadophora prunicola* sp. nov. **A–G** conidiophores and conidiogenous cells **H** conidia **A–H** from SNA**A–H**LM. Scale bar: 5 μm (**A** applies to **B–H**).

##### Description.

*Sexual morph* not observed. *Asexual morph on SNA. Vegetative mycelium* hyaline, septation hardly visible, branched, 1–3 µm wide, sometimes becoming brown with age, chlamydospores absent, hyphae of strain GLMC 735 in some parts inflated and restricted at the septae and up to 5 µm wide. *Sporulation* abundant, conidia formed on hyphal cells. *Conidiophores* hyaline, simple or septate and branched, up to 50 µm long. *Conidiogenous cells* enteroblastic, hyaline, smooth-walled, cylindrical, often inflated and bent in the upper part or attenuated at the base, delicate (disintegrating quickly), 4–15 × 1.5–2 µm, in strains GLMC 735 and GLMC 1574 sometimes integrated, necks cylindrical, 3–3.5 × 1 µm, collarettes distinct, funnel-shaped, cylindrical, 1–3 µm long, 1–2 µm wide at the upper edge, opening 1–1.5 µm wide, periclinal thickening sometimes observed. *Conidia* aggregated in heads, hyaline, smooth-walled, aseptate, ellipsoidal, cylindrical to ovoidal, straight, rarely slightly curved, both ends rounded, (2.5–)3–6.5(–9) × 1.5–2 µm, mean ± SD = 4.9 ± 1.8 × 1.7 ± 0.3 µm, L/W ratio = 2.8, conidia of strain GLMC 1574 smaller, measuring (2.5–)3.5–5(–6.5) × 1.5–2.5(–3) µm, mean ± SD = 4.2 ± 0.7 × 1.4 ± 0.4 µm, L/W ratio = 2.1.

##### Culture characteristics.

*Colonies on SNA* (strains GLMC 735, GLMC 1574 and GLMC 1633) flat with an entire to undulate margin, whitish, lacking aerial mycelium, reverse same colours, 18–27 mm diam. in 2 wk (25 °C in the dark). *Colonies on OA* (strains GLMC 735, GLMC 1574 and GLMC 1633) flat with an entire margin to undulate margin, buff, very pale luteus to cinnamon, lacking aerial mycelium, except for strain GLMC 1574 that was partly covered by white woolly aerial mycelium, reverse buff to fawn, 20–27 mm diam. in 2 wk (25 °C in the dark). *Colonies on PDA* (CBS 120891) flat to raised, entire margin, mycelium and surface white to very pale smoke-grey, with age turning isabelline to olivaceous in the centre, abundant velvety aerial mycelium, reverse straw to pure yellow, 18 mm diam. in 2 wk (25 °C). *Colonies on MEA* (CBS 120891) raised with entire margin, mycelium and surface white to very pale luteous, with age turning isabelline, abundant velvety aerial mycelium, reverse buff, honey to salmon, in diffuse daylight with a concentric apricot ring between centre and margin, 24 mm diam. in 2 wk (25 °C).

##### Notes.

*Cadophora prunicola* was isolated from *Prunus salicina* (2) in the Western Cape Province of South Africa, from *P. cerasus* (3) and *P. domestica* (2) in Saxony and *P. domestica* (3) in Baden-Württemberg, Germany. The strains from South Africa, as well as three strains from both hosts from Saxony and two strains from Baden-Württemberg, were selected for the phylogenetic analyses. This species is similar to *C. novi-eboraci* and *C. africana*, but differs by forming conidiophores of up to 50 µm length and conidiogenous cells that are often inflated. Subglobose or tear-shaped conidia as in *C. africana* have not been observed. The ITS, *TUB* and *EF-1α* sequences of *C. prunicola* differ in 8, 29 and 9 nucleotides, respectively, from *C. novi-eboraci* and in 11, 30 and 20 nucleotides, respectively, from *C. africana*.

A blastn search with the ITS sequence of *C. prunicola* in GenBank showed a 100% identity with an uncultured *Cadophora* from dead wood of *Fagus sylvatica* in Germany (LC015696, Floren et al. 2015).

##### Additional material examined.

Germany, Saxony, orchard east of Lungkwitz, 50°56'12.4"N, 13°47'36.6"E, from brown wedge-shaped necrosis in wood of *Prunus cerasus*, 11 Aug 2015, S. Bien leg., GLM-F106569, culture GLMC 735 = CBS 145521 = DSM 109135; Baden-Württemberg, orchard west of Nussbach, 48°31'55.8"N, 8°00'52.4"E, from brown necrosis in wood of *P. domestica*, 23 Aug 2016, S. Bien leg., GLM-F110714, culture GLMC 1633 = CBS 145522 = DSM 109146; Baden-Württemberg, orchard east of Nussbach, 48°31'57.3"N, 8°01'49.6"E, from brown wedge-shaped necrosis in wood of *P. domestica*, 23 Aug 2016, S. Bien leg., GLM-F110654, culture GLMC 1574; South Africa, Western Cape province, Franschhoek, from necrosis in wood of *P. salicina* close to old pruning wound, 10 June 2004, U. Damm leg., STE-U 6103.

#### Cadophora ramosa

Taxon classificationFungiChaetothyrialesHerpotrichiellaceae

S.Bien & Damm sp. nov.

95A8E1A2-D2C6-571B-9DE2-4405C9AF1A47

832110

Figures 5I , 13

Cadophora spadicis Travadon, D.P.Lawr., Roon.-Lath., Gubler, W.F.Wilcox, Rolsh. & K.Baumgartner, *Fungal Biology* 119(1): 62 (2015). nom. inval., Art. 40.6 (Shenzhen)(Synonym).

##### Type.

Germany, Saxony, orchard north of Kunnerwitz, 51°07'27.5"N, 14°56'36.3"E, from dark brown necrosis in wood of *Prunus cerasus*, 15 Jan 2015, S. Bien leg., GLM-F106227 – ***holotype***; GLMC 377 = CBS 145523 = DSM 109144 – culture ex-type.

##### Etymology.

Named after the often densely branched conidiophores (*ramosus* Lat. = branching).

**Figure 13.**
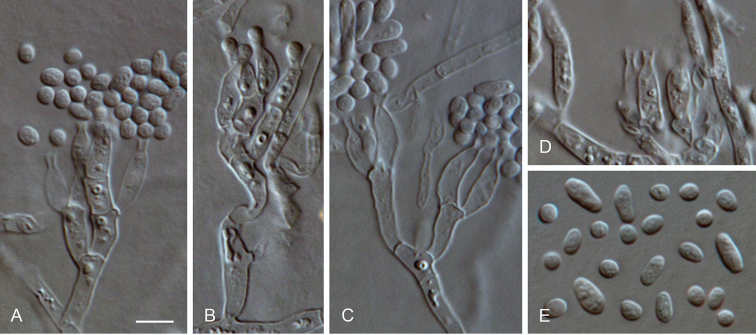
*Cadophora ramosa* sp. nov. **A–D** conidiophores and conidiogenous cells **E** conidia **A–E** from SNA**A–E**LM. Scale bar: 5 μm (**A** applies to **B–E**).

##### Description.

*Sexual morph* not observed. *Asexual morph on SNA. Vegetative mycelium* hyaline, smooth-walled, septate, branched, 1–5 µm wide, chlamydospores absent. *Sporulation* abundant, conidia formed on hyphal cells. *Conidiophores* hyaline, smooth-walled, septate, often densely branched, up to 50 µm long. *Conidiogenous cells* enteroblastic, hyaline, smooth-walled, flask-shaped, 4.5–11.5 × 2.5–3.5 µm µm, collarettes narrowly funnel-shaped, 1.5–2 µm long, 1–1.5 µm wide at the upper edge, opening 0.5–1 µm, periclinal thickening sometimes observed. *Conidia* aggregated in heads, hyaline, smooth-walled, aseptate, subglobose, ovoidal, ellipsoidal to elongate-ellipsoidal, straight, with both ends rounded, different spore-shapes formed from the same conidiogenous cells, sporulation often inside the medium, (3.5–)4–6(–9) × 2–2.5(–3) µm, mean ± SD = 4.9 ± 1.2 × 2.2 ± 0.3 µm, L/W ratio = 2.2, rarely up to 15 × 2.5 µm.

##### Culture characteristics.

*Colonies on SNA* flat with an entire margin, hyaline, filter paper partly pale olivaceous to olivaceous, lacking aerial mycelium, reverse same colours, 32–40 mm diam. in 2 wk (25 °C in the dark). *Colonies on OA* flat with an entire margin, pale cinnamon, with an umber inner and pale luteous outer margin, partly covered by woolly white to grey aerial mycelium, reverse pale cinnamon, with a citrine inner and pale luteous outer margin, 24–28 mm diam. in 2 wk (25 °C in the dark).

##### Notes.

*Cadophora ramosa* was previously described from grapevine in North America as *C. spadicis* (Travadon et al. 2015). Although Travadon et al. (2015) indicated *C. spadicis* as a new species, they listed a basionym and added the authorities of that basionym in brackets with the new name, as if they would combine an already existing species in a new genus, which was not the case. As Travadon et al. (2015) described *C. spadicis* as a new species, they should have listed a holotype (Art. 40.6, Art. 9.1, Turland et al. 2018); however, they listed a neotype, although original material was available (Art. 9.8). Therefore, the name *C. spadicis* is invalid. Moreover, the “neotype” listed is a living strain and not a (metabolically inactive) specimen. The species listed as “basionym” of *C. spadicis* by Travadon et al. (2015), *C. melinii*, was based on a wrong identification of strain CBS 111743 by Prodi et al. (2008), the strain that was listed as “neotype” of *C. spadicis*. However, the ex-type strain of *C. melinii*, CBS 268.33, was included in the study of Travadon et al. (2015) and belonged to a different clade in the phylogeny of that publication. Moreover, the authors listed as authorities of the “basionym” are the authors of the publication in which strain CBS 111743 was wrongly identified (Prodi et al. 2008) and not the authorities of *C. melinii*. Finally, although probably not intended as the whole name, prior to the authorities and “sp. nov.”, Travadon et al. (2015) listed “*Cadophora spadicis*CBS 111743”, which could be interpreted as not being a binary combination consisting of the name of the genus followed by a single specific epithet (Art. 23.1).

As the name *C. spadicis* is invalid, we described the species newly as *C. ramosa* on the basis of a specimen from *Prunus cerasus* in Saxony, Germany, collected in this study. The morphology of the ex-type strain of *C. ramosa* shows a high morphological concordance with the strains described as *C. spadicis* by Travadon et al. (2015). Conidiophores, conidiogenous cells, conidia and collarettes have similar shapes and sizes. The ITS, *TUB* and *EF-1α* sequences of *C. ramosa* differ at most in two, four and two nucleotides, respectively, which is a lower genetic variation than in *C. luteo-olivacea* and *C. novi-eboraci*.

#### Minutiella pruni-avium

Taxon classificationFungiPhaeomoniellalesPhaeomoniellaceae

S.Bien & Damm sp. nov.

9C4681DB-4ABD-54C9-83D4-2ABCD19AAEBE

832111

Figures 5J , 14

##### Type.

Germany, Baden-Württemberg, orchard west of Nussbach, 48°31'55.8"N, 8°00'52.4"E, from brown necrosis in wood of *Prunus avium*, 23 Aug 2016, S. Bien leg., GLM-F110704 – ***holotype***; GLMC 1624 = CBS 145513 = DSM 109150 – culture ex-type.

##### Etymology.

Name refers to the host species, *Prunus avium*.

**Figure 14.**
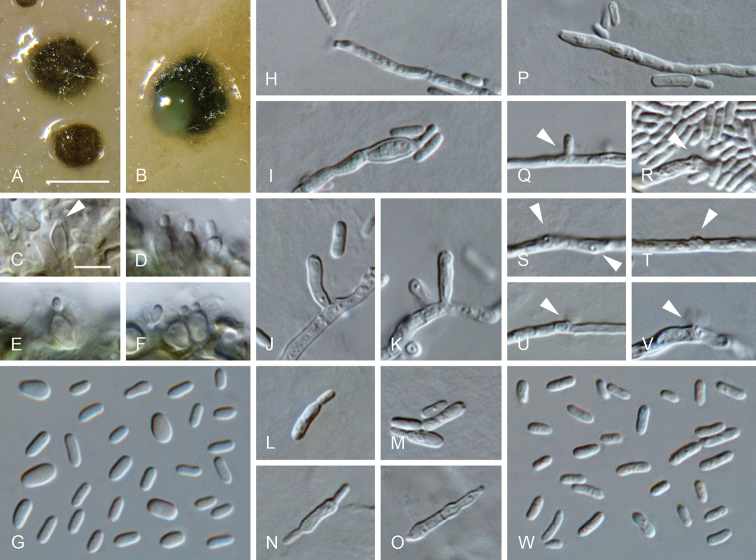
*Minutiella pruni-avium* sp. nov. **A, B** conidiomata **C–F** conidiogenous cells lining the inner wall of a conidioma **G** conidia formed in conidiomata **H–K, P–V** conidiogenous cells formed on hyphal cells (arrows indicate conidiogenous openings) **L–O** mother cells **W** conidia formed on hyphal cells **A–G** from OA**H–W** from SNA**A, B**SM, **C–W**LM. Scale bars: 200 μm (**A** applies to **B**), 5 μm (**C** applies to **D–W**).

##### Description.

*Sexual morph* not observed. *Asexual morph on SNA. Vegetative hyphae* hyaline, smooth-walled, septate, branched, 1–3 μm wide, lacking chlamydospores. *Sporulation* abundant, conidia formed directly on hyphal cells, in conidiomata and by microcyclic conidiation. *Conidiophores on hyphae* reduced to conidiogenous cells, conidiogenous loci formed terminally. *Conidiogenous cells* enteroblastic, hyaline, smooth-walled, mostly reduced to mere openings with collarettes formed directly on hyphal cells, discrete phialides rare, navicular, constricted at the base, 5.5–14.5 × 1.5–2.5 μm; collarettes rarely visible or flaring, < 0.5–3 μm long, opening 0.5–1.5 μm, periclinal thickening sometimes visible. *Conidia* aggregated in masses around the hyphae, hyaline, smooth-walled, aseptate, oblong to ellipsoidal, mostly straight, sometimes slightly curved, with both ends rounded, sometimes with a prominent scar on one end, (2.5–)3–5(–6) × 1–1.5(–2) μm, mean ± SD = 3.9 ± 0.9 × 1.4 ± 0.2 μm, L/W ratio= 2.8. *Conidiomata* produced on OA in 2–4 wk; solitary or aggregated, globose to subglobose, unilocular, immersed to superficial, 50–340 μm wide, olivaceous to black, mostly glabrous, sometimes with a few hairs, opening with an irregular rupture. *Conidiophores* reduced to conidiogenous cells. *Conidiogenus cells* enteroblastic, hyaline, smooth-walled, conidiogenous loci formed terminally, discrete phialides, globose to ampulliform or navicular, 3.5–7.5 × 2–3.5 μm, opening 0.5–1 μm, periclinal thickening sometimes visible. *Conidia* hyaline, smooth-walled, cylindrical to ellipsoidal, sometimes slightly curved, with both ends rounded, (2.5–)3–4.5(–6) × (1–)1.5–2(–3) μm, mean ± SD = 3.8 ± 0.8 × 1.7 ± 0.4 μm, L/W ratio = 2.2. *Microcyclic conidiation* occurs from minute collarettes at one or both ends of primary conidia that develop into swollen mother cells, often thick-walled, sometimes septate, > 5 μm long, 2–3.5 μm wide.

##### Culture characteristics.

*Colonies on OA* flat with entire margin, white to saffron, with scattered umber spots due to conidiomata formation, aerial mycelium lacking, spore masses oozing from conidiomata buff, reverse white to buff, 4–8 mm diam. in 2 wk, 6–10 mm diam. in 4 wk. *Colonies on SNA* flat with entire margin, white, lacking aerial mycelium, reverse same colour; < 1–2 mm diam. in 2 wk, 6–8 mm diam. in 4 wk.

##### Notes.

Two strains of *Minutiella pruni-avium* were isolated from wood of *Prunus avium*. The LSU sequences of these strains differ in three and one nucleotides from those of *M. tardicola* and *Minutiella* sp., respectively. The ITS region shows 11 differences to *M. tardicola* and 9 differences to *Minutiella* sp. The *TUB* sequence of *M. tardicola* and *Minutiella* sp. differ in one nucleotide, however, in 35 and 33 nucleotides compared to *M. pruni-avium*. *Minutiella pruni-avium* differs from *Minutiella tardicola* and the strains of *Minutiella* sp. by forming larger conidiomata, longer discrete phialides and flaring collarettes of up to 3 μm.

The closest match in a blastn search with the ITS sequence of strain GLMC 1624 is the type strain of *Minutiella tardicola*CBS 121757 with 97.9% identity (NR132006, Damm et al. 2010).

##### Additional material examined.

Germany, Baden-Württemberg, orchard west of Nussbach, 48°32'11.3"N, 8°01'01.3"E, from brown necrosis in wood of *Prunus avium*, 23 Aug 2016, S. Bien leg., GLM-F110750, culture GLMC 1667 = CBS 145514 = DSM 109149.

#### Proliferodiscus ingens

Taxon classificationFungiHelotialesHyaloscyphaceae

S.Bien & Damm sp. nov.

72D1C306-4238-5066-BA4D-98F1E1B1303B

832112

Figures 5K , 15

##### Type.

Germany, Baden-Württemberg, orchard south of Oppenau, on a hill, 48°27'57.6"N, 8°09'11.0"E, from necrotic wood of *Prunus avium*, 24 Aug 2016, S. Bien leg., GLM-F110834 – ***holotype***; GLMC 1751 = CBS 145519 = DSM 109148 – culture ex-type.

##### Etymology.

Named after the comparatively huge conidiomata (*ingens* Lat. = huge).

**Figure 15.**
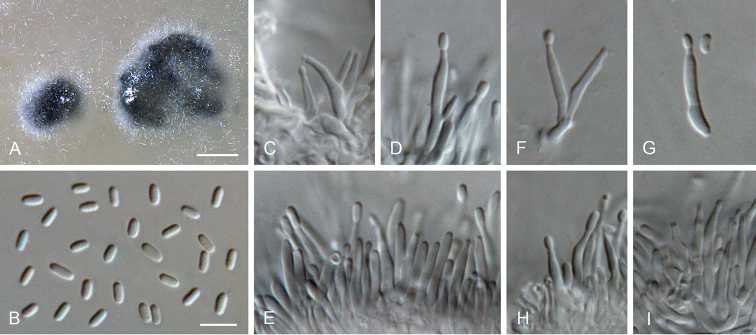
*Proliferodiscus ingens* sp. nov. **A** conidiomata **B** conidia formed in conidiomata **C–E, H, I** conidiogenous cells lining the inner wall of a conidioma **F, G** detached conidiogenous cells **A–I** from OA**A**SM**B–I**LM. Scale bars: 300 μm (**A**), 5 μm (**B** applies to **C–I**).

##### Description.

*Sexual morph* not observed. *Asexual morph on OA. Vegetative hyphae* hyaline, smooth-walled, septate, branched, 1.5–3 μm wide, lacking chlamydospores. *Sporulation* abundant, conidia formed in conidiomata. *Conidiomata* produced on OA in 2–4 wk, solitary or aggregated, subglobose, unilocular, superficial, 250–1000 μm wide, dull green to grey olivaceous, almost glabrous to completely covered with hairs, opening with an irregular rupture. *Conidiophores* hyaline, smooth-walled, septate, sometimes branched at the base and above, conidiogenous loci formed terminally. *Conidiogenus cells* enteroblastic, hyaline, smooth-walled, navicular to subulate, tapering towards apices, 8–15 × 1–2 μm; collarettes hardly visible, cylindrical, < 1 μm long, opening 0.5–1 μm, periclinal thickening sometimes visible. *Conidia* hyaline, smooth-walled, aseptate, cylindrical to ellipsoidal, straight, with both ends rounded, 2.5–3(–3.5) × 1–1.5 μm, mean ± SD = 2.9 ± 0.2 × 1.4 ± 0.1 μm, L/W ratio = 2.1.

##### Culture characteristics.

*Colonies on OA* raised with entire to crenated margin, buff to pale olivaceous grey, white at the margin, with umber to black spots due to conidiomata, aerial mycelium sparse, white, reverse buff to cinnamon, 1–2 mm diam. in 2 wk, 2–3 mm diam. in 4 wk. *Colonies on SNA* flat to very low convex with entire to fimbriate margin, white, lacking aerial mycelium, reverse same colour; 1–2 mm diam. in 2 wk, 2–3 mm diam. in 4 wk.

##### Notes.

Strain GLMC 1751, described here as *Proliferodiscus ingens*, was isolated from *Prunus avium* in Baden-Württemberg. Only the asexual morph of this fungus was observed. Asexual morphs have previously rarely been observed in the genus *Proliferodiscus* and no complete description is available. However, Baral and Kriegelsteiner (1985) and Dennis (1949) mention an asexual morph of *Pr. pulveraceus*. Dennis (1949) observed multilocular pycnidia with slender conidiophores (10–12 μm long) and spherical conidia (1 μm diam.), whereas Baral and Kriegelsteiner (1985) described oval conidia, measuring 1.5–1.7 × 1.2–1.4 μm, produced on verticillately branched conidiophores. In contrast to the description of Dennis (1949), the strains observed here produce unilocular pycnidia. Conidia of *Pr. ingens* are larger than conidia of *Pr. pulveraceus* in both descriptions. The asexual morph of *Pr. ingens* differs from that of the other *Proliferodiscus* strains observed in this study by producing larger, darker conidiomata, a different conidial shape and a lower growth rate.

The closest match in a blastn search with the ITS sequence of strain GLMC 1751 with 97.7% identity is “Hyaloscyphaceae sp. 2” strain ICMP 18979 from symptomless leaves of *Nothofagus fusca* in New Zealand (JN225935, Johnston et al. 2012).

#### Proliferodiscus sp.

Taxon classificationFungiHelotialesHyaloscyphaceae

791C323D-83BE-53F6-BDE3-AB1D84DA51E9

Figures 5L , 16

##### Description.

*Sexual morph* not observed. *Asexual morph on OA. Vegetative hyphae* hyaline, smooth-walled, septate, branched, 1.5–3 μm wide, lacking chlamydospores. *Sporulation* abundant, conidia formed in conidiomata. *Conidiomata* produced on OA, SNA and pine needles in 2–4 wk, solitary or aggregated, subglobose, unilocular, superficial, 125–500 μm wide, luteous, almost glabrous to completely covered with hairs, opening with an irregular rupture. *Conidiophores* hyaline, smooth-walled, septate, simple or branched, conidiogenous loci formed terminally. *Conidiogenus cells* enteroblastic, hyaline, smooth-walled, navicular to subulate, tapering towards apices, 9–14 × 1–2 μm, collarettes cylindrical, < 1 μm long, opening 0.5–1 μm, periclinal thickening sometimes visible, conidiogenous cells often extend to form new conidiogenous openings, extensions flask-shaped to navicular. *Conidia* hyaline, smooth-walled, aseptate, mostly globose to obovoid, sometimes cylindrical to ellipsoidal, straight, with both ends rounded, 1.5–2(–3) × 1.5(–2) μm, mean ± SD = 1.9 ± 0.3 × 1.5 ± 0.1 μm, L/W ratio = 1.2.

**Figure 16.**
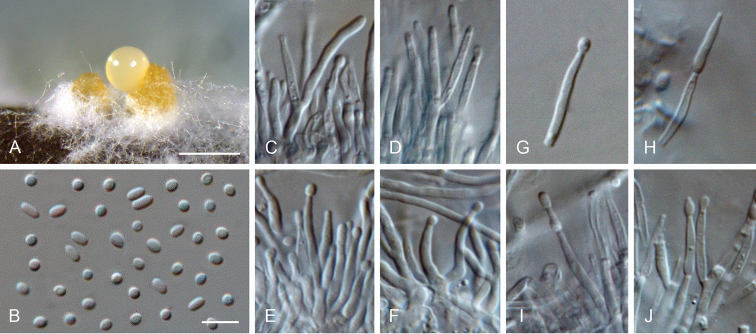
*Proliferodiscus* sp. **A** conidiomata **B** conidia formed in conidiomata **C–F, I–J** conidiogenous cells lining the inner wall of a conidioma **G, H** detached conidiogenous cells **H–J** extensions on conidiogenous cells **A–J** from OA**A**SM**B–J**LM. Scale bars: 300 μm (**A**), 5 μm (**B** applies to **C–J**).

##### Culture characteristics.

*Colonies on OA* flat to effuse with entire to fimbriate margin, white to buff, cinnamon to sienna at the margin, aerial mycelium sparse, white, reverse buff, cinnamon to sienna; 6–14 mm diam. in 2 wk, 16–32 mm diam. in 4 wk. *Colonies on SNA* flat to effuse with entire to fimbriate margin, white, lacking aerial mycelium, reverse same colour; 6–18 mm diam. in 2 wk, 20–34 mm diam. in 4 wk.

##### Notes.

In total, five strains of *Proliferodiscus* sp. have been isolated from wood of *Prunus domestica* in Saxony (3) and Lower Saxony (1) as well as *P. avium* in Baden-Württemberg (1). Two subclades are formed by these strains, which differ in one and four nucleotides in the LSU and ITS sequences, respectively. No morphological differences were noticed between strains of the two subclades. They form a well-supported clade (100/100/99%) with eight strains retrieved from GenBank, including the ex-type strain of the recently described *Pr. chiangraiensis*. The conidial shape of these strains is similar to that of the asexual morph of *Pr. pulveraceus* observed by Baral and Kriegelsteiner (1985); the conidia are slightly larger, but the measurements overlap. However, we cannot link these strains to *Pr. pulveraceus* on this basis, because the species was described based on its sexual morph and no sequences of type material are available. Moreover, a recently published sequence, designated as *Pr. pulveraceus* (MN066320, G Marson unpubl.), belongs to a different clade in our phylogeny.

One striking feature was observed in our collections: new conidiogenous cells grow out of conidiogenous openings (Fig. 16H–J). This feature has previously been observed in species of other genera, for example, *Fusarium graminearum* (Domsch et al. 2007) and several *Colletotrichum* species (Damm et al. 2012, 2019).

The anamorphic states of the observed strains of *Proliferodiscus* sp. differ from *Pr. ingens* (strain GLMC 1751, this study) by the colour and the smaller size of conidiomata, faster culture growth rate on OA and SNA and the shape of the conidia.

##### Material examined.

Germany, Saxony, in orchard north of Wölkau, 50°58'42.3"N, 13°49'40.0"E, from brown necrosis in wood of *Prunus domestica*, 16 Jan 2015, S. Bien leg., GLM-F106310, culture GLMC 460 = CBS 145517 = DSM 109138; Baden-Württemberg, orchard west of Nussbach, 48°31'55.8"N 8°00'52.4"E, from necrotic wood of *P. avium*, 23 Aug 2016, S. Bien leg., GLM-F110844, culture GLMC 1761 = CBS 145518 = DSM 109137; Saxony, in orchard north of Wölkau, 50°58'42.3"N, 13°49'40.0"E, from brown necrosis in wood of *P. domestica*, 16 Jan 2015, S. Bien leg., GLM-F106320, culture GLMC 470; Lower Saxony, Hollern-Twielenfleth, orchard, 53°36'13.6"N, 9°31'50.8"E, from brown wedge-shaped necrosis in wood of *P. domestica*, 8 Oct 2015, S. Bien leg., GLM-F107151, culture GLMC 1301; Saxony, in orchard north of Wölkau, 50°58'42.3"N, 13°49'40.0"E, from brown necrosis in wood of *P. domestica*, 16 Jan 2015, S. Bien leg., GLM-F106352, culture GLMC 502.

## Discussion

The new genus *Arboricolonus* is described, based on one strain, GLMC 459, that could not be assigned to any known genus. Closest matches of ITS (90% identity) and LSU (97% identity) sequences of this fungus with strains identified at least to the genus level were strains of *Glutinomyces vulgaris*, *Chalara aurea*, *Hyalodendriella betulae* and *Polyphilus sieberi*; (Ashrafi et al. 2018; Crous et al. 2007; Nakamura et al. 2018; Vu et al. 2019). The asexual morph of *Arboricolonus simplex* clearly differs from these genera. The monotypic genus *Hyalodendriella* forms pigmented micro- and macroconidiophores directly on hyphae as well as pigmented limoniform to ellipsoid and prominently apiculate conidia (Crous et al. 2007), while *Chalara* is characterised by forming sessile or stalked phialides with basal ventres, long collarettes and deep-seated conidiogenous loci; conidia are cylindrical and often produced in basipetal chains (Holubová-Jechová 1984; Kowalski 2006). However, *Chalara*, is highly polyphyletic; species have been placed in different classes of Ascomycota (Paulin and Harrington 2000; Koukol 2011). As the type species belongs to the Sordariomycetes (*Chalara fusidioides*), strain CBS 880.73 probably does not even belong in *Chalara* s. str. The genera *Polyphilus* and *Glutinomyces* were described, based on sequence data and colony morphology only (Ashrafi et al. 2018; Crous et al. 2017; Nakamura et al. 2018); no asexual morphs are available that could be compared to *Arboricolonus*.

For systematic placement of the genus on order level, we conducted a class-wide phylogenetic analysis of LSU-ITS with reference sequences of Leotiomycetes which clearly places it in the order Helotiales (data not shown). The closest matches from LSU and ITS blastn searches indicate the relationship of this new genus to the Hyaloscyphaceae, the largest family of the Helotiales that is mainly circumscribed by features of sexual morphs (Jaklitsch et al. 2016). Han et al. (2014b) and Johnston et al. (2019) demonstrated the polyphyly of the family. For the placement of the new genus on family level, we included LSU and ITS sequences of selected sequences from all clades of Hyaloscyphaceae and closely related families recognised by Han et al. (2014b) in their multi-locus ML analyses, as well as the closest matches from the blastn searches. The clades in our phylogeny are mostly well-supported and in agreement with the clades in Han et al. (2014b). However, most of these clades are placed on a polytomous branch. Due to the lack of a stable backbone, the exact placement of clades in relation to each other shown by the ML analyses of Han et al. (2014b) could not be confirmed and remains inconclusive. Furthermore, as shown in Fig. 1, family designation of the included strains according to Han et al. (2014b), Johnston et al. (2019) and Ekanayaka et al. (2019) is highly problematic.

The new genus *Arboricolonus* clusters in our phylogeny with sequences of *Polyphilus*, *Cistella*, *Rodwayella* and *Polydesmia*, however, on short branches, they lack posterior probability or bootstrap support. Therefore, we consider the placement of the genus as of uncertain taxonomic position on family level (Helotiales, incertae sedis). We did not find any record of asexual morphs of *Cistella* and *Rodwayella* for morphological comparison with the new genus. In contrast to *Arboricolonus simplex*, *Polydesmia pruinosa* (asexual morph: *Brefeldochium pruinosum*) produces septate falcate conidia in sporodochia (Verkley 2005).

Except for the lack of microcyclic conidiation, the genus *Arboricolonus* morphologically resembles *Collophorina* and related genera by forming slow growing cultures, conidiophores that are reduced to short phialides or openings on hyphae with minute to flaring collarettes and cylindrical to ellipsoid conidia with obtuse ends (Bien et al. 2020). A similar morphological appearance could be explained with a similar lifestyle within plant wood and is therefore regarded as a result of convergent evolution. The possibility of morphological adaptation of collophorina-like species to the habitat within the woody plant body has previously been discussed by Bien et al. (2020).

In total, 29 strains of *Cadophora* have been isolated from wood in Germany, all from *Prunus cerasus* and *P. domestica*, of which 17 were included our phylogeny. A further three strains included in the analyses originated from wood of *P. salicina* in South Africa.

The strains of *C. novi-eboraci* from this study were all isolated from wood of *P. cerasus* in Saxony and Bavaria, Germany. *Cadophora novi-eboraci* was described from decaying wood of *Vitis* spp. in the USA (Travadon et al. 2015) and recently reported from necrotic wood of *Malus domestica* in Germany (Gierl and Fischer 2017). To our knowledge, this is the first report of *C. novi-eboraci* from *Prunus* wood worldwide.

Strain CBS 101359 from stained wood of *Actinidia chinensis* in Italy had been referred to as *C. malorum* by Di Marco et al. (2004) and Prodi et al. (2008). Travadon et al. (2015) identified it as *C. novi-eboraci*. In our phylogeny, it is placed distantly from both species. We therefore regard this strain as a different taxon.

*Cadophora luteo-olivacea* was originally isolated from wastewater in Sweden (Van Beyma 1940). This species has been reported mostly from *Vitis vinifera* and several other woody hosts worldwide (Farr and Rossman 2019), but not from *Prunus*. Fischer et al. (2016) isolated this species from grapevine nurseries and vineyards in Germany. In this study, *C. luteo-olivacea* was isolated from *Prunus domestica* in all three sampling areas. Therefore, this is the first report of *C. luteo-olivacea* from *Prunus* wood worldwide. *Cadophora luteo-olivacea* seems to be not only widely distributed, but also very variable. Gramaje et al. (2011) observed a high variability of colony pigmentation within *C. luteo-olivacea*, which was also observed by Harrington and McNew (2003), not only in this, but also in other species of the genus. We noticed that the *TUB* and *EF-1α* sequences of the ex-type strain CBS 141.41 and strain A42, identified as *C. luteo-olivacea* by Travadon et al. (2015), differed in 8 and 16 nucleotides, respectively. Strains, isolated from *Prunus* wood in this study, show up to nine and five nucleotide differences in the *TUB* and *EF-1α* sequences, respectively. In the resulting single-locus trees (not shown), subgroups are formed that are, however, not concordant and therefore do not represent independent evolutionary lineages. This phenomenon was previously studied in the highly variable species *Colletotrichum siamense* (Liu et al. 2016), that had been regarded as species complex, based on single-locus analyses. Except for a small cluster formed by two strains from the study of Travadon et al. (2015), no subgroups are formed in the multi-locus phylogeny.

Three of the *Cadophora* species we isolated from *Prunus* wood, namely *C. luteo-olivacea*, *C. novi-eboraci* and the newly described *C. ramosa* (syn. *C. spadicis*), were previously associated with wood diseases like cankers or Petri disease of *Vitis* spp., (e.g. Casieri et al. 2009; Halleen et al. 2007; Fischer et al. 2016; Travadon et al. 2015; Pintos et al. 2018). Several other fungal species are reported both from *Vitis* and fruit trees as well, for example, several species belonging to the Botryosphaeriaceae, Diatrypaceae and the genus *Phaeoacremonium* (Damm et al. 2007, 2008; Moyo et al. 2018); fruit trees were referred to as alternative hosts of grapevine trunk disease pathogens. Similar to these fungi, *Cadophora* species could be transferred to grapevine plants from *Prunus* trees grown in close vicinity to vineyards. To our knowledge, the genus *Cadophora* has never been reported from *Prunus* before (Farr and Rossman 2019 as well as all references listed in this study). Moreover, we found new species on this host genus. One of them, *C. prunicola*, was isolated from three different *Prunus* species, *P. cerasus*, *P. domestica* and *P. salicina*, both in Germany and South Africa. A second new species, *C. africana*, is so far only known from *P. salicina* in South Africa. Based on a blastn search with its ITS sequence, *C. prunicola* was detected as an uncultured *Cadophora* in dead wood of *Fagus sylvatica* in Germany (Floren et al. 2015), but so far, there is no report of any of these two species in *Vitis* wood.

In addition to the strains isolated from *Prunus* trees in Germany and South Africa, we included strains of *Phialophora bubakii*, because we noticed a close affinity to the genus *Cadophora* by preliminary sequence comparisons (not shown). *Phialophora bubakii* that was originally described from margarine as *Margarinomyces bubakii* (Laxa 1930) and combined in *Cadophora* in this study, had previously been reported from wood of *Pinus strobus* and *Populus* sp. (Ellis 1976), where it caused blue stain on timber, from *Betula pendula* in Poland (Mulenko et al. 2008), further from subcutaneous infections (Porto 1979) and from corneal ulcers (Eiferman et al. 1983), both in humans and dogs in several countries (Hoog et al. 2000). An ITS sequence of a strain from wood pulp of *Populus tremula*, identical to that of the ex-isotype strain, is available in GenBank (Vu et al. 2019), confirming the occurrence of *Ph. bubakii* on *Populus* wood. The remaining reports lack sequence data and are therefore doubtful. Some of the reports could actually refer to other species that could have been confused due to similar morphology. None of the *C. bubakii* and *C. obscura* strains, included in this study, originated directly from wood or infections of mammals. However, the ex-isotype strain of *C. obscura* apparently originates from water in a “trämassefabrik” (trämassa = wood pulp) and therefore possibly from the processed wood itself. An identical ITS sequence from archaeological wood in Greenland (NB Pedersen et al., unpubl. data) suggests the occurrence of *C. obscura* in wood as well.

The *TUB* sequences of *C. bubakii*, *C. obscura* and *C. viticola* were excluded from the phylogenetic analyses, because all of them differed tremendously from each other and from the rest of the dataset. Furthermore, sequencing *TUB* of *C. obscura* (CBS 269.33), using either the forward or the reverse primer, generated two vastly differing sequences (data not shown). Sequencing *TUB* of *Aspergillus* spp. by Peterson (2008) and Hubka and Kolarik (2012) also resulted in different sequences from the same species, which were regarded as possible paralogous gene fragments. Based on the data in this study, we assume a similar situation in *Cadophora*.

All *Cadophora* species treated in this study can be distinguished by all single loci analysed (data not shown). Due to the high genetic variation within some of them, the use of more than one locus is recommended for further studies on this genus.

The genus *Minutiella* was isolated for the first time from wood of *Prunus armeniaca* in South Africa and described as *Phaeomoniella tardicola* (= *M. tardicola*) (Damm et al. 2010). This is the first report of the genus *Minutiella* and the Phaeomoniellales, in general, from *P. avium* and *P. domestica* worldwide. The genus *Minutiella* is, so far, only known from wood of *Prunus* trees. More specifically, *M. tardicola* is known from *P. armeniaca* in South Africa, the new species *M. pruni-avium* from *P. avium* in Germany and *Minutiella* sp. from *P. domestica* in Germany (Damm et al. 2010; this study). This genus also forms reduced conidiogenous cells; probably an adaption to the living conditions inside wood like *Collophorina* and related species and *Arboricolonus* (Bien et al. 2020; this study).

The two *Minutiella* strains GLMC 1636 and GLMC 1687 are morphologically indistinguishable from *M. tardicola*, however, differ in LSU, ITS and *TUB* sequences from this species. The description of this further new species is in preparation (C. Kraus, pers. comm.).

The LSU-ITS-*TUB* phylogeny of the Celotheliaceae shows a high similarity to the previously compiled LSU phylogeny in Chen et al. (2015). In this study, we provide the first multi-locus phylogeny of the family. For a conclusive placement of genera within this family, more data is needed, since all phylogenies lack a deep node support (Chen et al. 2015; this study). “*Phaeomoniella*” *pinifoliorum* apparently represents a separate genus. A new genus was, however, not described, as no strain and only ITS sequence data were available for characterisation of this genus.

*Proliferodiscus* has been reported from wood and bark of several woody hosts worldwide (Albertini and Schweinitz 1805; Dennis 1949; Baral and Kriegelsteiner 1985; Spooner 1987; Weber and Bresinsky 1992; Haelewaters et al. 2018; Farr and Rossman 2019). Dennis (1949) lists *Prunus insititia* as one of the hosts of *Proliferodiscus pulveraceus*. In this study, two *Proliferodiscus* species have been collected from wood of *P. avium* and *P. domestica*.

The species delimitation in the genus *Proliferodiscus* was previously based on the morphology of the sexual morphs only (e.g. Haines and Dumont 1983; Baral and Kriegelsteiner 1985; Spooner 1987; Hofton et al. 2009). Six of the species had been described even before 1900 in other genera and were transferred to this genus later. Only few morphological treatments of *Proliferodiscus* species contained information on asexual morphs (Dennis 1949; Baral and Kriegelsteiner 1985). The genus has not been treated in modern terms yet.

There are sequences of ten strains/specimens identified as *Proliferodiscus* in GenBank, none of them is ex-type, except for the recently described *Pr. chiangraiensis*. The available sequences of *Proliferodiscus* belong to three main clades in our phylogeny. One well-supported clade in our phylogeny contains several apparently closely related strains/specimens, for which different names have been applied.

Most of our strains from *Prunus*, belonging to two subclades of the same main clade, did not show any morphological differences of the asexual morph and only differed in few nucleotides from each other and from the remaining specimens/sequences within this clade. Therefore, we refrained from describing two new species in this clade and refer to the strains as *Proliferodiscus* sp. In order to allow comparison with asexual morphs in this genus in the future, we provided a description of this species, as well as of the newly described species *Pr. ingens*. In order to provide a solid basis for identifications and detections of new species, *Proliferodiscus* species need to be epitypified and data of both sexual and asexual morphs, as well as sequence data, need to be provided.

## Conclusion

The isolation of fungal strains from necrotic wood of *Prunus* species in Germany and South Africa revealed several unknown taxa within Leotiomycetes and Eurotiomycetes. Based on morphology and multi-locus molecular analyses, we described one new genus and six new species in four genera. Although previously unknown from wood of *Prunus* trees, the genus *Cadophora* was revealed to be a common wood inhabitant of *P. cerasus* and *P. domestica* in Germany, but apparently not of *P. avium*. The genus *Minutiella*, originally described from *P. armeniaca* in South Africa, also occurs in *Prunus* wood in Germany and, thus, belongs to the common genera in *Prunus* wood as well. Our analyses of the genus *Proliferodiscus* also contributes to the knowledge of this genus by the first detailed descriptions of asexual morphs of this genus. The results underline the sparse knowledge of several fungal genera from wood and of the wood mycobiome of the economically important host genus *Prunus*. The morphological data presented here and the up-to date molecular frameworks will provide a basis for further studies on these genera and on wood diseases of *Prunus* trees.

## Supplementary Material

XML Treatment for Arboricolonus

XML Treatment for Arboricolonus simplex

XML Treatment for Cadophora africana

XML Treatment for Cadophora bubakii

XML Treatment for Cadophora luteo-olivacea

XML Treatment for Cadophora novi-eboraci

XML Treatment for Cadophora obscura

XML Treatment for Cadophora prunicola

XML Treatment for Cadophora ramosa

XML Treatment for Minutiella pruni-avium

XML Treatment for Proliferodiscus ingens

XML Treatment for Proliferodiscus

## Supplementary materials

Supplementary material 1 Complete list of strains included in this study, with collection details, GenBank accession numbers and references Link: https://doi.org/10.3897/mycokeys.63.46836.suppl1 Data type: molecular data https://binary.pensoft.net/file/385051This dataset is made available under the Open Database License (http://opendatacommons.org/licenses/odbl/1.0/). The Open Database License (ODbL) is a license agreement intended to allow users to freely share, modify, and use this Dataset while maintaining this same freedom for others, provided that the original source and author(s) are credited.Steffen Bien, Ulrike Damm
