# Corrigendum: Bien S, Damm U (2020) *Arboricolonus simplex* gen. et sp. nov. and novelties in *Cadophora*, *Minutiella* and *Proliferodiscus* from *Prunus* wood in Germany. MycoKeys 63: 163–172. https://doi.org/10.3897/mycokeys.63.46836

**DOI:** 10.3897/mycokeys.69.55264

**Published:** 2020-07-14

**Authors:** Steffen Bien, Ulrike Damm

**Affiliations:** 1 Senckenberg Museum of Natural History Görlitz, PF 300 154, 02806 Görlitz, Germany Senckenberg Museum of Natural History Görlitz Germany; 2 International Institute Zittau, Technische Universität Dresden, Markt 23, 02763 Zittau, Germany Senckenberg Museum of Natural History Görlitz Germany

## Abstract

In the original article the herbarium code of the Senckenberg Mu­seum of Natural History Görlitz, Germany, was wrongly cited as GLMC. The correct herbarium code of the institution is GLM.

We combined *Margarinomyces bubakii* in the genus *Cadophora*. This was not compliant with the International Code of Nomen­clature for algae, fungi, and plants article F.5.1 (Turland et al. 2018), because a registration identifier was lacking, which is compulsory since 1 January 2013. The combination is now validated by providing the MycoBank number below.

In the original article, the herbarium code of the Senckenberg Museum of Natural History Görlitz, Germany, was wrongly cited as GLMC. The correct herbarium code of the institution is GLM.

We combined *Margarinomyces bubakii* in the genus *Cadophora*. This was not compliant with the International Code of Nomenclature for algae, fungi and plants article F.5.1 (Turland et al. 2018), because a registration identifier was lacking, which is compulsory since 1 January 2013. The combination is now validated by providing the MycoBank number below.

## Taxonomy

### Cadophora bubakii

Taxon classificationFungiHelotialesPloettnerulaceae

(Laxa) Damm & S.Bien comb. nov.

23901652-4B45-546A-AA8F-F04273CF87BC

MycoBank No: 835799

Margarinomyces bubakii Laxa, Centralbl. Bakteriol. 2. Abth. 81: 392. 1930. (Basionym)
≡ Phialophora bubakii (Laxa) Schol-Schwarz, Persoonia 6 (1): 66. 1970.

## Supplementary Material

XML Treatment for Cadophora bubakii
